# Spontaneous buckling of contractile poroelastic actomyosin sheets

**DOI:** 10.1038/s41467-018-04829-x

**Published:** 2018-06-25

**Authors:** Y. Ideses, V. Erukhimovitch, R. Brand, D. Jourdain, J. Salmeron Hernandez, U. R. Gabinet, S. A. Safran, K. Kruse, A. Bernheim-Groswasser

**Affiliations:** 1 Department of Chemical Engineering, Ilse Kats Institute for Nanoscale Science and Technology, Ben Gurion University of the Negev, Beer-Sheva, 84105 Israel; 2 Department of Theoretical Physics, Universität des Saarlandes, Postfach 151150, Saarbrücken, 66041 Germany; 3 NCCR Chemical Biology, Departments of Biochemistry and Theoretical Physics, University of Geneva, 1211 Geneva, Switzerland; 4 Department of Chemical and Biological Physic, Weizmann Institute of Science, Rehovot, 76100 Israel

## Abstract

Shape transitions in developing organisms can be driven by active stresses, notably, active contractility generated by myosin motors. The mechanisms generating tissue folding are typically studied in epithelia. There, the interaction between cells is also coupled to an elastic substrate, presenting a major difficulty for studying contraction induced folding. Here we study the contraction and buckling of active, initially homogeneous, thin elastic actomyosin networks isolated from bounding surfaces. The network behaves as a poroelastic material, where a flow of fluid is generated during contraction. Contraction starts at the system boundaries, proceeds into the bulk, and eventually leads to spontaneous buckling of the sheet at the periphery. The buckling instability resulted from system self-organization and from the spontaneous emergence of density gradients driven by the active contractility. The buckling wavelength increases linearly with sheet thickness. Our system offers a well-controlled way to study mechanically induced, spontaneous shape transitions in active matter.

## Abstract (web summary)

Active matter composed of filaments and molecular motors can contract. Here, the authors report the spontaneous out-of-plane buckling of reconstituted contracting poroelastic actomyosin sheets in the absence of external cues.

## Introduction

Contracting materials have many potential applications related to artificial muscles, sensing of mechanical stimuli, and active self-assembly of three-dimensional structures^1,2^. In particular, two-dimensional intrinsically contractile sheets may find applications as actively functional band-aids that speed up wound healing or in form of beating patches that could support heart function. Artificial contractile materials are often based on synthetic molecular motors^3^ or use liquid crystals^1^ that require external cues such as electrical stimuli, light, or temperature for activation and control. However, tremendous challenges must be overcome before synthetic active materials can be used for human health. An alternative approach is to employ reconstituted biological materials, notably viscous or elastic acto-myosin networks similar to those found in living tissues. In addition to being obviously biocompatible, such systems demonstrate spontaneous contractility and self-organization^4–7^. The characteristics of contraction can be readily controlled by changing global parameters, such as the concentration of molecular motors or of passive cross-linkers^4–7^. In addition, contraction patterns and dynamics can be regulated by spatially patterned activation of myosin motors^8,9^.

Contracting materials are part of essentially all biological organisms. They allow cells to sense mechanical properties of the environment^10^ or enable deformations that can be used to deform the organism, which could be exploited for morphogenetic processes during development^11^. In these cases, buckling has been observed when a contractile epithelium was subjected to anisotropic contraction^12^ and/or coupled to an elastic substrate^13^. How intrinsic contractions in absence of coupling to a substrate can intrinsically generate three-dimensional structures, however, has not yet been explored.

In fact, in developing biological systems, the formation of folds and other three-dimensional structures from two-dimensional objects is often attributed to in-plane growth^14–20^. However, there are systems, where structure formation occurs in absence of growth, for example in hornbean leaves^21^ or during gastrulation of animal embryos^11,13,22,23^.

These examples suggest that spontaneously contractile materials could promote out-of-plane deformations of sheets. Currently, however, we lack good model systems for fundamental studies of such phenomena. A notable exception is provided by thin, elastic chemically crosslinked gel sheets that show large-scale buckling upon non-uniform lateral contraction^24^. However, in this case, spatial variations in contraction result from pre-imposed gradients in the cross-linker density. Furthermore, in many cases—naturally occurring or artificial—contractile sheets are coupled to a substrate or other elastic structures^8,9,11,20–23^, presenting an additional difficulty for studying contraction induced folding.

Here, we demonstrate the fabrication and dynamical properties of initially homogenous, thin, suspended, elastic acto-myosin sheets of controllable extent and elastic properties. These gel sheets spontaneously contract through the activity of myosin motors with no need for external stimuli save for the presence of ATP in solution. Even more remarkable, the gel sheets can self-organize into folded structures: buckles appear spontaneously in the absence of externally applied mechanical constraints and without imposed spatial variations of the gel composition or mechanical properties. The characteristic length scale of the folded pattern is proportional to the gel thickness and can thus be easily controlled. By analyzing the solvent flow during the gel contraction dynamics, we show that the acto-myosin gel behaves as a poroelastic active material. Using a theory of actively contractile, poroelastic gels, extended to include the catch-bond behavior of myosin motors, we explain the observations and the physical basis of the contraction dynamics. Overall, our results show that buckling can be spontaneously generated by motor activity and does not require mechanical coupling to the environment or pre-imposed gradients in sheet material properties.

## Results

### Formation of thin contractile actin sheets

**Fig. 1:**
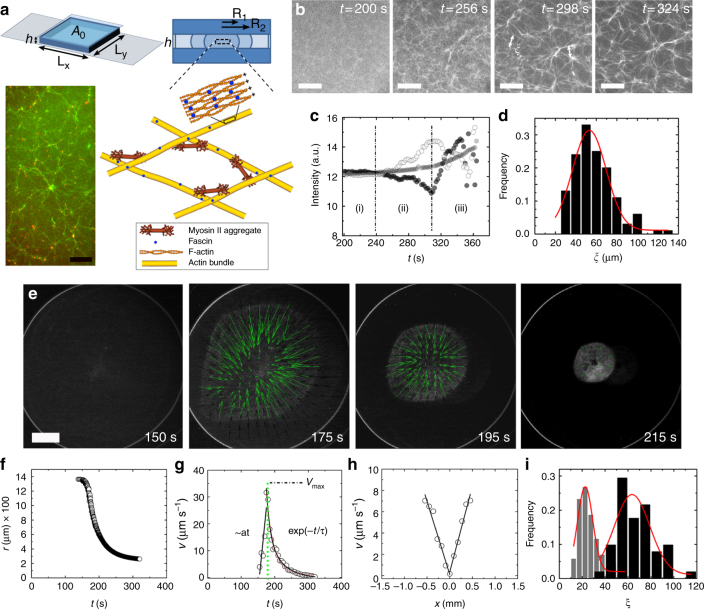
Spontaneous formation and contraction of elastic acto-myosin sheets. **a** Sketch of experimental cell chambers of height *h* and lateral extensions *L*_*x*_ and *L*_*y*_. Partial filling generates sheets of various radii. Fluorescence micrograph of an actomyosin network at contraction onset (*t* = 285 s): actin (green) and myosin aggregates (red) showing actin bundles crosslinked by motor aggregates as illustrated in the schematic. **b** Fluorescence micrographs of a polymerizing and actively reorganizing actomyosin network. Actin network reorganization is visible for *t* > 240 s, contraction starts at *t* = 308 s. The arrows at *t* = 298 s illustrate the mesh size. **c** Fluorescence intensity at three different points for the gel in **b**. Phase (i) represents homogenous actin polymerization and bundle formation. Phase (ii) corresponds to the reorganization of filament bundles by myosin motors, which leads to local accumulation of actin at some points and dilution at others. During phase (iii), the gel contracts macroscopically. White and black circles represent the actin densities in a small region of 40 μm in diameter (local information), gray circles in a large region of 600 μm in diameter (global information). **d** Distribution of mesh size for the gel in **b** at contraction onset *ξ*_0_ (*t* = 308 s) based on *N*_meshes_ = 75 meshes. **e** Fluorescence images of actin obtained from a top view of a contracting circular gel. Green arrows indicate the gel contraction velocity field obtained from PIV. Chamber height *h* = 150 μm, initial gel radius *R* = 1.38 mm, and drop volume 0.9 μL. **f**–**h** Gel radius (**f**) and contraction velocity (**g**) as a function of time, and contraction velocity profile at *t* = 210s (**h**) for the gel in **e**. **i** Distribution of mesh sizes for the gel shown in **a**. Mesh size distribution at contraction onset (*t* = 285 s, black columns, *N*_meshes_ = 55) and at advanced stages of contraction (*t* = 370 s, gray columns, *N*_meshes_ = 90). Scale bars: 100 μm (**a**), 100 μm (**b**), 500 μm (**e**); actin was labeled with Alexa-Fluor 488, myosin with Alexa-Fluor 568

Internally driven contractile gels were generated by polymerizing actin in the presence of the strong passive cross-linker fascin and clusters of myosin II motors^5,25^ (Fig. 1a, Methods). Only if actin polymerization occurred in the presence of motors and cross-linkers, contractile elastic gels were produced where the motors were embedded in the network^5,7,25^. To generate thin sheets, we simultaneously introduced actin monomers, myosin, and fascin in chambers with a large aspect ratio $\sqrt {A_0} /h$, where *A*_0_ is the chamber area in the *x*- and *y*-directions and *h* the height in the *z*-direction (Fig. 1a). The chamber surfaces were treated to avoid sticking of the actin gel (Methods). After adding a drop of the protein mixture, the chamber was sealed with a cover glass. The chambers were only partially filled to avoid sticking of the gel to the lateral boundaries. In this way, a circular symmetric drop was obtained. Its size depended on the chamber height, which varied between 70 and 250 μm, and the drop volume, which was between 0.3 and 7 μL. Typically, the drops had initial radii *R* of 0.12–0.5 cm.

After mixing 5 M G-actin with 16.7 nM myosin motors and 280 nM fascin, defining the time *t* = 0, the system evolved in three phases (Fig. 1b, Supplementary Movie 1). In the first phase, which lasted 4 min, actin filaments nucleated and polymerized spontaneously. The protein distribution remained homogenous down to scales of several μm as one can infer directly from the fluorescence images and from the actin and myosin pair correlation functions *G*(*r*) (Fig. 1b, c, Supplementary Figure 1a–d, Methods). Note that concomitant with actin polymerization, the distribution of motors, while remaining homogenous, coarsens (Supplementary Figure 1e, f). In the second phase, which lasted 1 min, the system evolved into an interconnected network of actin bundles if both, fascin and myosin clusters, were present^5,25^ (Fig. 1a, b). The network remained macroscopically homogenous and isotropic (Fig. 1b, c gray dots) with a mean mesh size *ξ*_0_ = 60 ± 16 μm (mean ± SD, *N*_meshes_ = 75; *N*_gels_ = 1) at the end of this phase (Fig. 1d). This property is also reflected by the pair correlation function *G*(*r*) (Supplementary Figure 2). After this phase, the myosin motors dominantly localized at intersection points of filament bundles (Fig. 1a). In the third phase, the gel started to contract (Fig. 1b, c). The existence of this phase required prior formation of filament bundles that are mechanically sufficiently stable and a connection of these bundles by myosin motor clusters^5,7,25^. Consequently, the velocity of myosin clusters was the same as the local gel displacement velocity (Supplementary Figure 3, Supplementary Movie 2). Note that the three phases were probably not mutually exclusive as network reorganization could still have been accompanied by actin polymerization.

Contraction was isotropic throughout the process. This is in line with observations made on contracting actin gels that are attached at the periphery, where motors have been activated by light in specific areas and where it was shown that the final state presents the same aspect ratio as the initial state^9^. In the course of contraction, the radius *r*, which is determined from the projected gel area *A*_gel_, $r = \sqrt {A_{{\mathrm{gel}}}/\pi }$, decreased by 80%, such that *r*_final_ = 0.2*R* (Fig. 1e, f, Supplementary Figure 5, Supplementary Movie 3). The contraction phase can be divided into two sub-phases: in the beginning, the edge velocity $v = \frac{{{\mathrm d}r}}{{{\mathrm d}t}}$ increased approximately linearly with time until it reached its maximal value *v*_max_ at time *t*_max_ (Fig. 1g). For times later than *t*_max_, the edge velocity decayed to zero exponentially with a characteristic time *τ* = 20 s. As particle image velocimetry (PIV) analysis shows, contraction always began at the gel boundaries (Fig. 1e, Supplementary Movie 3). The local gel contraction velocity decreased linearly from the gel boundary towards the gel center (Fig. 1h). During contraction, the network mesh size decreased (Fig. 1i), whereas the filament bundles remained straight, as confirmed by comparing the end-to-end distance *l*_end-to-end_ and contour length *l*_cont_ of filament bundles (Supplementary Figure 4c). This is different from contracting, dilute actin networks, where the weak cross-linker α-actinin was used instead of the strong cross-linker fascin and where individual actin filaments buckled^26,27^. Since our network started contracting from the boundary, the mesh size increased from the boundary to the center of the gel corresponding to decreasing gel volume fraction in that direction (Supplementary Figure 4). For an initially homogenous sheet, this contraction mode resulted in an increase of the gel density first at the periphery (Supplementary Figure 5f–i) and only later propagated into the gel interior (Supplementary Figure 5j). The density increase occurred gradually until it saturated at late times (Supplementary Figure 5j). In the stationary state, the contractile forces and the gel elasticity were equilibrated and the gel was homogenous again.

### Contracting actin sheets can spontaneously buckle

**Fig. 2:**
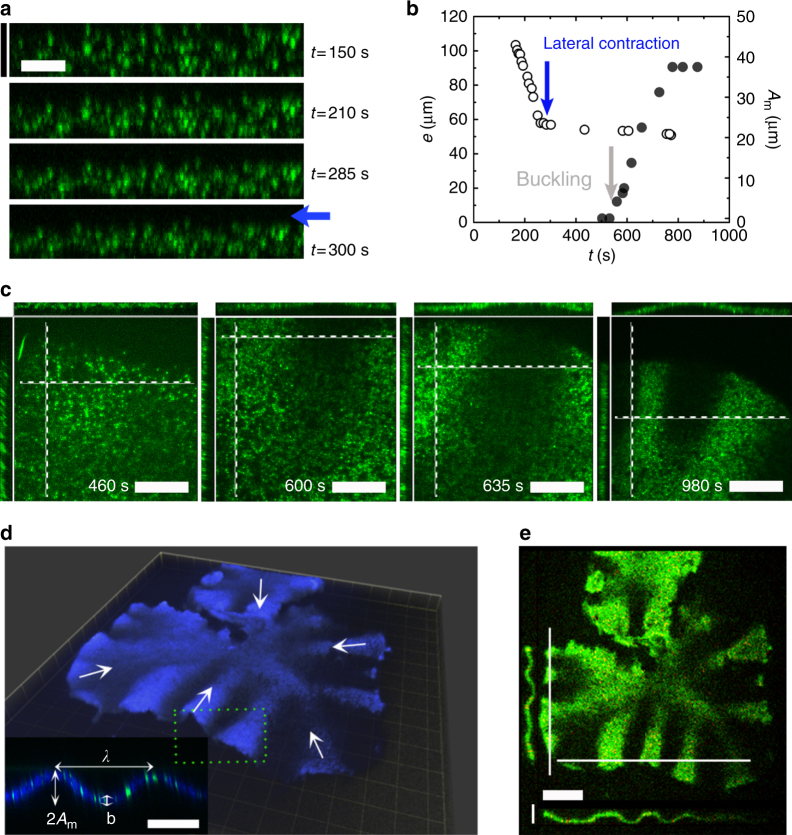
Buckling of an acto-myosin sheet. **a** Spinning disk confocal fluorescence micrographs of actin in the *xz* plane of a contracting gel. The blue arrow marks the time when lateral contraction sets in and indicates the direction of contraction. Vertical scale bar: 100 μm, horizontal scale bar: 50 μm. **b** Evolution of gel height *e* and buckling amplitude *A*_m_ for the gel in **a**. The blue arrow marks the time when lateral contraction sets in and the gray arrow marks the time when the sheet starts to buckle at the periphery. **c** Spinning disk confocal fluorescence micrographs of the actin gel in **a** in the *xy* plane (top view), the *xz* plane (top side view), and *yz* plane (left side view). Side views (*xz* and *yz* planes) are measured along the white dotted lines. Scale bar: 180 μm. **d** Laser scanning confocal micrograph of a three-dimensional view of a buckling gel at steady state. Inset: side view of the gel indicating the wavelength *λ*, final gel thickness *b*, and buckling amplitude *A*_m_. Scale bar: 100 μm. **e** Top view (*xy* plane) and side views (*xz* and *yz* planes) along the white lines of the gel in **d**. Horizontal scale bar: 200 μm. Vertical scale bar: 80 μm. Drop volume is 4.9 μL (**a**–**c**) and 4.6 μL (**d**, **e**). Chamber height *h* = 160 μm (**a**–**c**), *h* = 80 μm (**d**, **e**). Initial gel radius *R* = 3.1 mm (**a**–**c**), *R* = 4.3 mm (**d**, **e**). Actin was labeled with Alexa-Fluor 488 (green in **a**, **c**, **e**, blue in **d**), myosin was labeled with Alexa-Fluor 568 (green in **d**, red in **e**). **d**, **e** Same gel as in ref. ^5^ Fig. 7f

We now turn to the dynamics along the short axis, the vertical *z*-direction. Similar to its lateral contraction, the gel contracted from an initial thickness *e* of 160 µm (the chamber height) to about 50 µm (Fig. 2a, Supplementary Movie 4). The vertical contraction began earlier than the lateral one with a faster relaxation of the vertical contraction velocity, *τ*_z_/*τ* ≈ 1/3 (Supplementary Figure 6). Lateral contraction started only after the vertical contraction was essentially completed: during lateral contraction, the gel thickness *e* decreased slightly from 57 to 52 µm (Fig. 2b). This corresponds to an effective Poisson ratio *ν* ≈ 0. At advanced stages of planar contraction, the gel spontaneously started to buckle at about 580 s which corresponds to 1.5*τ* after *t*_max_ (Supplementary Figure 6), as is evident from the developing bright and dark stripes that emerge perpendicularly to the boundary (Fig. 2c, Supplementary Figure 7, Supplementary Movie 5). The emerging folds were directed perpendicularly to the boundary (Fig. 2d, white arrows) and had a roughly sinusoidal shape (Fig. 2d, e, Supplementary Movie 6). We characterize this structure by means of “wavelengths”, i.e., the distances between adjacent intensity maxima and adjacent intensity minima. The steady state thickness of that gel was *b* = 23.5 ± 3.4 μm (mean ± SD, *N*_thicknesses_ = 69 for *N*_gels_ = 1, Methods) and was uniform throughout the gel (Fig. 2e). We determined the average wavelength of the buckled state by measuring the distances between adjacent folds. To this end we used a confocal slice and analyzed the fluorescence intensity along a line tracing the border of the gel (Supplementary Figure 8, Methods). Analyzing the gel profile in the *xz* and *yz* planes close to the gel boundary gave very similar values (Fig. 2d, e). The distribution of wavelengths of the folds had an average value of *λ* = 196 ± 33 μm (mean ± SD, *N*_wavelengths_ = 24 for *N*_gels_ = 1. Supplementary Figure 8). In two-dimensional cuts across the *xy* plane, the folds appeared as areas of increased and decreased fluorescence intensity (Fig. 2e). The resulting folds were visible as straight bright strips at low magnification (Supplementary Figure 9).

### The contracting actomyosin sheet is a poroelastic gel

To obtain a better understanding of the system dynamics, we now characterize the material properties of the system taking into account the actin gel, the myosin motors, and the aqueous phase within the gel. It has been proposed that actin gels in living cells behave as poroelastic materials^28–30^. There are two distinguishing features of such materials^28^: First, the generation of a flow of the penetrating solvent caused by active myosin contractility. Second, diffusive stress relaxation characterized by an effective diffusion constant that depends on the drained elastic modulus, the solvent viscosity, and the gel porosity.

**Fig. 3:**
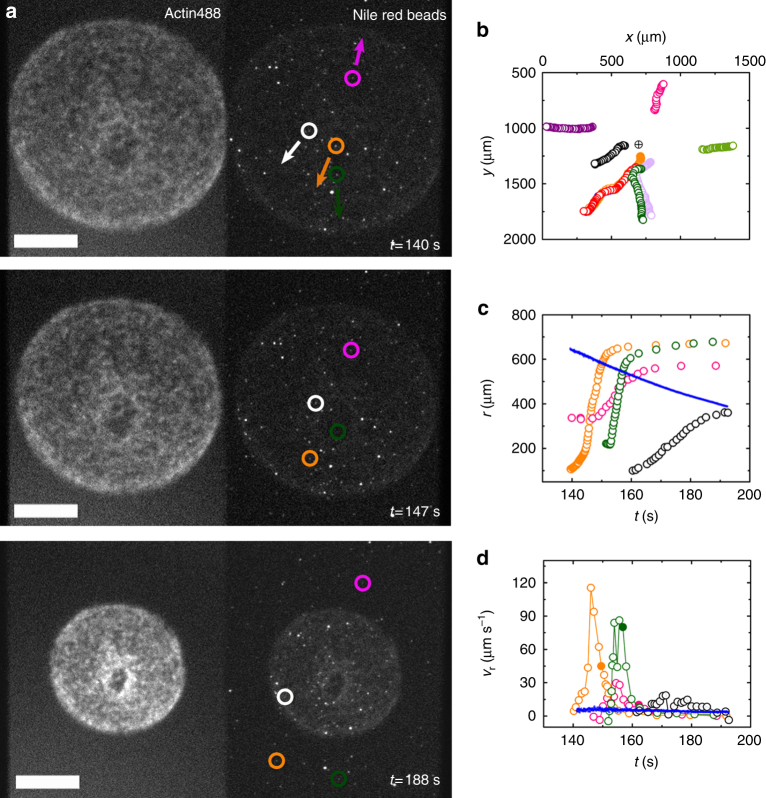
Acto-myosin sheets are poroelastic I. **a** Subsequent epifluorescence micrographs of a contracting gel with embedded fluorescent beads with a diameter of 2300 nm at low magnification. Circles mark the positions of four beads shown in **b**–**d**. Arrows mark the global direction of beads motion. Actin was labeled with Alexa-Fluor 488, beads were Nile red. Scale bar: 400 μm. **b** Trajectories of nine selected beads. Encircled cross: center of the gel (*x*_0_, *y*_0_). **c** Radial distance from the gel center of four beads (open circles) and gel radius (blue dots) as a function of time (Methods). **d** Radial velocity *v*_r_ of the same beads as in **c** (open circles) and of the gel edge (blue dots) (Methods). The filled circles indicate the time, when the beads left the gel. For clarity, in **a**, **c**, **d** only a subset of the beads shown in **b** are presented. Drop volume is 1.0 μL, chamber height *h* = 140 μm, and initial gel radius *R* = 1.5 mm

We first checked whether myosin contractility generated a solvent flow. To measure the solvent flow, we added fluorescent beads to the solution. We used beads of 2.3 μm in diameter during the initial and intermediate phases of contraction, when the average pore size was larger than 15 μm, and beads of 200 nm diameter in the later contraction phase, when the pore size was smaller. The fluorescent beads, on average, moved in the outward radial direction as the gel contracted toward its center (Fig. 3a, b, Supplementary Movie 7). The beads’ radial velocity *v*_r_ was up to 20 times larger than the gel edge velocity as long as they were inside the gel (Fig. 3c, d, Supplementary Figure 10, Methods). As the beads moved outwards from the gel center, their velocities initially increased dramatically by more than an order of magnitude. This was consistent with the gel contraction velocity profile, which also increased from the cell center to the periphery (Fig. 1h). The beads slowed down as they approached the boundary, where the gel densified. The filled circles in Fig. 3d indicate the time, when the beads left the gel. Outside the gel, the beads still moved for some time. Note that this movement was not due to inertia, because the Reynold number is less than 2 × 10^−4^ even for the largest bead velocity of 100 μm s^−1^ and bead size of 2.3 μm. For later times, the average velocity of the beads decreased and fluctuated (Fig. 3d).

**Fig. 4:**
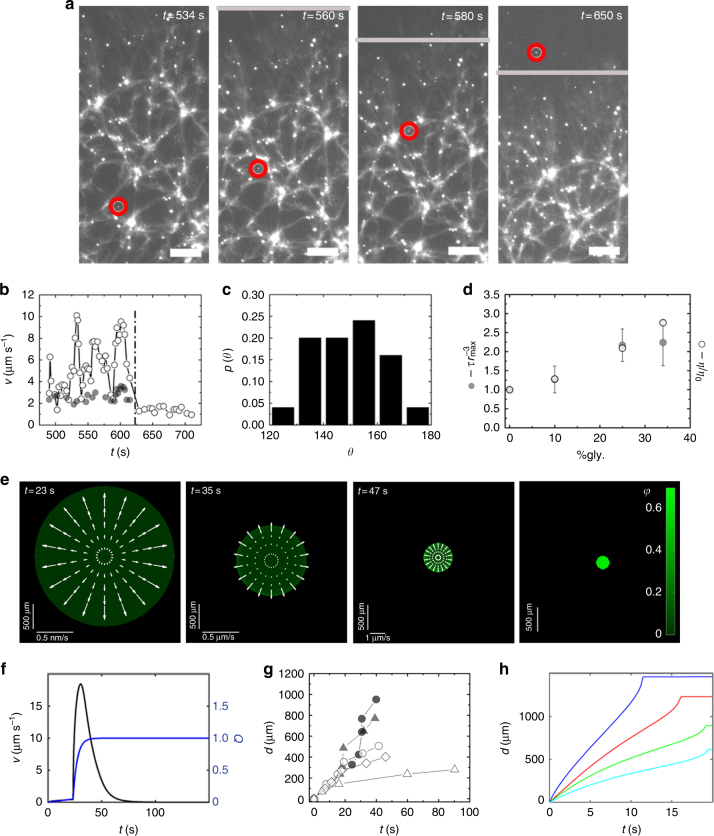
Acto-myosin sheets are poroelastic II. **a** Subsequent epifluorescence micrographs of a contracting gel with embedded fluorescent beads with a diameter of 200 nm at high magnification. Red circle marks one bead in subsequent micrographs. Gray line indicates the gel boundary. Actin is labeled with Alexa-Fluor 488. Beads are Fluoresbrite Yellow-Green. Scale bars: 100 μm. **b** Local speed of the gel (gray dots) and of the bead (white circles) at nearby positions in **a**. Dashed line represents the time, when the bead left the gel. **c** Distribution of angles *θ* between the local gel and bead velocities from *N*_pairs_ = 25 velocity pairs. **d** Solvent viscosity *η* and relaxation time *τ* divided by $r_{{\mathrm{max}}}^3$ as a function of glycerol percentage in the solvent. Quantities are normalized to their values at 0% glycerol. For each % of glycerol values are averaged over *N*_gels_ = 3 gels with initial radii of *R* = 1.4 mm and height *h* = 140 μm (drop volume is 0.87 μL). Error bars indicate standard deviation of experimental values. **e** Numerical solution to the dynamic equations. Green: gel volume fraction *φ*, arrows: solvent velocity field. Parameters are given in Supplementary Table 1. **f** Gel contraction speed *v* and fraction of bound motors *Q* as a function of time for the solution in **e**. **g** The distance *d* traveled by the density from the gel boundary inwards in the reference frame of the laboratory as a function of time for the gel shown in Fig. 5. Black dots, gray triangles, hollow circles, hollow diamonds, and hollow triangles correspond to increasing values of the density. Black dots and gray triangles correspond to time courses that start before *t* = *t*_max_. **h** As in **g** for the solution of the dynamic equations shown in **f** for volume fractions 0.007 (blue), 0.034 (red), 0.151 (green), and 0.271 (cyan)

We attributed the fluctuations in the bead velocities to the porous structure of the actin network. To test this hypothesis, we investigated with higher resolution bead trajectories towards the end of the contraction process, when all movements had slowed down. Indeed, the trajectories of the beads were tortuous (Fig. 4a, Supplementary Figure 11) and their velocities fluctuated between 2 and 10 µm s^−1^ (Fig. 4b). The bead velocities increased with their distance from the actin bundles (Supplementary Figure 12). Hence, the beads moved fastest in the center of a pore and slowest, when they were close to a bundle reflecting the local structure of the gel. In this advanced phase of gel contraction, the local solvent speed was on average faster than the local gel contraction velocity by a factor of 2.5 ± 0.7. The gel velocity-solvent velocity correlation function shows that locally, the fluid flow was always opposite to the gel flow (Fig. 4c, Methods). Again, the bead speed increased from the gel center towards the gel boundary (Supplementary Figure 13). All this indicates that as the gel contracted, the solvent was driven out as expected for a poroelastic material.

We now turn to the second property of poroelastic systems, namely that stress relaxation is determined by an effective poroelastic diffusion constant *D*. Explicitly, the relaxation time scales as $r_{{\mathrm{max}}}^2/D$, where $r_{{\mathrm{max}}}$ is the system radius at the beginning of the relaxation process, that is, at the time when the gel contraction velocity has reached its maximum. The value of *D* is proportional to an effective, concentration-dependent elastic modulus $\kappa$ of the gel and inversely proportional to an effective friction constant *γ* that accounts for the permeation of water through the actin network, such that $D \sim \kappa /\gamma$ (Supplementary Notes). The friction coefficient $\gamma$ scales as $\eta /\xi ^2$, where $\eta$ is the solvent viscosity and $\xi$ scales with the mesh size of the gel—i.e., the distance between crosslinks in the actin gel. The elastic modulus has units of energy per unit volume and is inversely proportional to the volume of a pore, whereas the friction coefficient *γ* depends on the pore facet perpendicular to the solvent motion. For the actin network, the pore size can be approximated by the mesh size. Initially, the gel is isotropic. Yet, at the time the lateral contraction velocity reached its maximum, the contraction in the *z*-direction was already essentially completed as mentioned above (Fig. 2b). Thus, the distance between two crosslinks of filament bundles is $\xi _\parallel$ within the contraction plane and $\xi _ \bot$ perpendicular to it, consequently, $\kappa \sim 1/\xi _\parallel ^2\xi _ \bot$ and $\gamma \sim \eta /\xi _{||}\xi _ \bot$^31,32^. Again, we take the values of these quantities at the time when the contraction velocity has reached its maximum, so that $\xi _\parallel \sim r_{{\mathrm{max}}}$. Altogether, we thus have $\tau \sim \eta r_{{\mathrm{max}}}^3$.

To test the dependence of the relaxation time on the solvent viscosity, we changed the latter by adding various amounts of glycerol. We conducted three experiments for each amount of glycerol. With increasing values of the viscosity, the duration of the polymerization and of the reorganization phases were extended (Supplementary Figure 14). In contrast, the linear acceleration and maximal contraction velocity did not change significantly (Supplementary Figure 14), suggesting that network organization during the polymerization and the reorganization phases adapts to changes in the solvent viscosity. The measured relaxation times obeyed the scaling relation derived above (Fig. 4d) further confirming the poroelastic nature of our system.

Having shown that the system is poroelastic, we can formulate a physical description of the contraction dynamics within the framework of active generalized hydrodynamics^33,34^. The system state is given in terms of the gel and solvent densities. Their time evolution is determined by the continuity equations that describe mass conservation of the two phases (Supplementary Notes). The respective gel and solvent velocity fields that appear in the continuity equations are obtained by balancing the forces due to gradients in the total mechanical stress in the gel with the friction forces resulting from motion of the actin gel relative to the surrounding fluid:

1 $$\gamma \left( {\partial _tu - v} \right) = \nabla \cdot \left( {\sigma ^{{\mathrm{el}}} + \sigma ^{{\mathrm{act}}}} \right).$$

Here, *u* denotes the displacement field of the actin gel and *v* is the solvent’s velocity field. Furthermore, *σ*^el^ is the drained elastic stress of the gel, which depends on the drained bulk and shear elastic constants *K* and *μ*, respectively (Supplementary Notes). The embedded motors generate an active contractile stress *σ*^act^. Within the framework of active generalized hydrodynamics it is expressed as −*Qζ*Δ*μ*II^35^. In this expression, Δ*μ* is the difference between the chemical potentials of ATP and its hydrolysis products, which drives the contraction process. The factor *ζ* is a phenomenological constant linking the active processes to the causing thermodynamic force Δ*μ*. In general*, ζ* depends on the filament and motor densities. We take it to be constant and account for the density of crosslinking motors through the fraction *Q* of bound motor molecules. Equivalently, the magnitude of the active stress is determined by the active force dipoles, generated by the molecular motors^36^, and is given by the force dipole density. We assume the active stress to be macroscopically isotropic and thus multiply its expression by the identity operator II. Equation (1) together with mass conservation (gel plus solvent) and the Stokes equation for the solvent describe the contraction of an active poroelastic gel (Supplementary Notes). This approach is simpler than those taken in refs. ^8,37^, where a nonlinear dependence of the stress on the filament or myosin densities, respectively, were considered for contracting cytoskeletal networks. In ref. ^9^, a static spring network without shear elasticity was used to describe contraction. Furthermore, all these works neglected the solvent dynamics, which we have shown above to be important at least in our system.

We next hypothesized that the acceleration phase of the contraction dynamics was due to changes in the fraction of bound myosin motors that increases with the mechanical stresses in the gel. In terms of force dipole stresses, this means that the force dipole moment changes. Indeed, myosin forms catch bonds^38^ that are strengthened in a macroscopically contracting network, because the actin filaments in-between the contractile myosin clusters (that act on filaments of opposite orientation) are stretched during network contraction. The fraction of bound motors *Q* is thus determined by

2 $$\partial _tQ = k_{{\mathrm{on}}}\left( {1 - Q} \right) - k_{{\mathrm{off}}}\left( {\sigma ^{{\mathrm{el}}}} \right)Q.$$

The rates *k*_on_ and *k*_off_, respectively, denote the myosin attachment and detachment rates. Whereas we take *k*_on_ to be independent of the elastic stress, the detachment rate *k*_off_ decreases with increasing elastic stress of the actin network *σ*^el^ reflecting the motor’s catch-bond characteristic according to Bell’s law^39^ (Supplementary Notes).

We solved the dynamic equations numerically using a finite difference scheme (Supplementary Notes). We use the experimental parameter values estimated from the relaxation dynamics and the buckling of the contractile sheets, see below. Notably, we use an effective elastic modulus of 0.05 Pa. For determining the friction coefficient between the solvent and the gel, we use a mesh size *ξ*_||_, *ξ*_⊥_ = 1200 μm^2^ taken from the experiment in Fig. 1b at *t*_max_, where we took *ξ*_⊥_ = *ξ*_*||*_/3 (taken from experiments, see below), and an effective viscosity of 0.05 Pa·s. This value accounts for molecules dissolved in the penetrating solvent that increase the viscosity with respect to pure water. In our numerical calculations, we mimicked the polymerization and reorganization phases by increasing the fraction *Q* of bound motors from 0 to *Q*_0_ linearly in time. During this phase, *Q* is kept spatially homogenous. We took *Q*_0_ = 0.15 as estimated in ref. ^5^. Afterwards, the value of *Q* evolved according to Eq. (2).

Our numerical solution of the dynamic equations for an initially homogenous, circular symmetric, elastic gel disc with an initial radius of 1.5 mm represented a radial contraction of the gel that starts from the boundaries (Fig. 4e). Contraction from the boundary was a consequence of the initially homogenous distribution of motor-induced force dipoles, which generated a homogenous active stress, such that active forces canceled each other everywhere but at the boundaries. The gel volume fraction was always highest at the boundary and decreased toward the gel center, until, in the final state, the gel density was again homogenous throughout the gel (Supplementary Figure 15). In the final state, the gel radius was 150 μm corresponding to a final lateral strain of 90%. Similar to the gel volume fraction, the elastic stress was also highest at the gel boundary but was uniform toward the end of the contraction process (Supplementary Figure 16). In the final state, mechanical equilibrium was reached as the effective stress diffusion current vanishes and the uniform elastic stress balances the uniform active stress. During the contraction process, the solvent was squeezed out of the gel at a velocity that increased with the distance from the gel center (Fig. 4e). The edge velocity of the contracting gel increases approximately linearly during the initial polymerization and reorganization phases, where the fraction of bound myosin *Q* was increased but remained approximately spatially homogenous (Fig. 4f). Subsequently, contraction accelerated dramatically until it reached a maximal velocity of about 20 μm s^−1^ and then relaxed exponentially in time toward 0 (Fig. 4f). The acceleration concurred with a strong increase in the fraction of bound motors, which is due to the catch-bond behavior (Fig. 4f). Throughout the contraction process, the fraction of bound motors remained essentially spatially homogenous (Supplementary Figure 17). This is due to the fact that the motor binding and unbinding rates are large compared to the time-scale of contraction and because we chose the motor unbinding rate to react very sensitively to stresses. The contraction velocity profile changed between the acceleration and the relaxation phases (Supplementary Figure 18); during the acceleration phase it was concave. It exhibited a convex part after the maximal contraction velocity was reached. The profile of the solvent velocity showed a similar change, however, it was delayed with respect to the change of the gel’s contraction velocity profile. In the late stages of the contraction process, the fluid speed exceeded the gel contraction speed (Supplementary Figure 18) similarly to what was measured experimentally (Figs. 3d and 4b, Supplementary Figures 10, 12). The good quantitative agreement between the experiments and the numerical results points to the physics underlying the observed behavior: contraction starts from the periphery as the active forces are initially imbalanced only at the boundaries, the contraction velocity increases due to the increased stress in the gel and the motors’ catch-bond characteristics, and poroelastic diffusion of the elastic stress yields eventually a uniform state of mechanical equilibrium.

Next, we looked at how the contraction of the gel propagated towards the gel center. To this end we chose to follow a value of the gel density as it propagates from the gel boundary inwards. The distance *d* traveled by the density in the reference frame of the laboratory shows different time dependence before and after *t*_max_ (Fig. 4g). Before *t*_max_, it is concave showing that the velocity at which it moves inwards increases. After *t*_max_, the curve is convex corresponding to a slowing down of this velocity. For higher values of the density, the time to reach the center increases even though the distance that needs to be traversed decreases. In the numerical solutions, we find the same dependence of the time needed to reach the center on the initial distance to the center as in the experiments. However, the dependence of the distance travelled on time is always convex in the numerical solutions suggesting that nonlinear elastic properties that are not captured by our description affect the dynamics of contracting actin gels (Fig. 4h).

### Estimation of the buckling wavelength

**Fig. 5:**
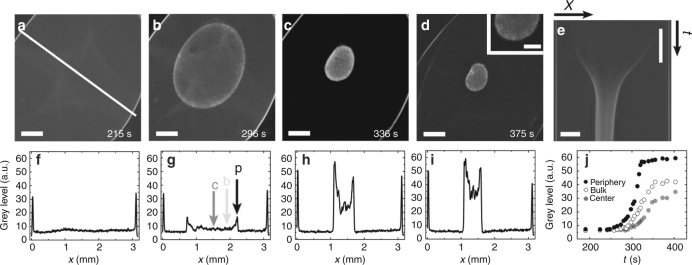
Density fields of buckling contracting sheets. **a**–**d** Subsequent epifluorescence micrographs of a buckling contracting sheet at low magnification. The buckles appear as straight bright strips at the gel periphery (**d**). Inset (**d**): zoom in at the bottom of the gel showing buckling. Scale bar: 200 μm. **e** Kymograph of the density along the line indicated in **a**. **f**–**i** Density profiles along the same line corresponding to **a**–**d**. **j** Density as a function of time for the points indicated in **g**. Actin is labeled with Alexa-Fluor 488. Scale bars: 500 μm (**a**–**e**). Time scale bar: 1 min (**e**). Drop volume is 1.1 μL, chamber height *h* = 140 μm, and initial gel radius of short and long axis: *R* = 1.2 and 2.1 mm

Having established the poroelastic nature of our reconstituted actin gels, we further investigated the buckling instability. In Fig. 5, we present the contraction (Fig. 5a–e) and the spatiotemporal evolution of the density (Fig. 5f–j) of a gel that buckles. Compared to gels that did not buckle (Supplementary Figure 5), the increase of the gel density at the periphery was relatively larger and persisted in steady state. These two situations differed furthermore in that the density gradients in buckling gels were steeper than in non-buckling gels. In both cases, the density increased first at the periphery (Fig. 5a, b, f, g). In the case of buckling, at some point, the density at the periphery saturated, whereas the bulk density continued to increase (Fig. 5h–j, Supplementary Movie 9). In this case, contraction proceeded apparently in presence of a fixed perimeter, which is only possible if the sheet buckles. This is reminiscent of minimal surfaces under the constraint of a fixed perimeter that is longer than the perimeter of a circle of the same surface area. In very rare cases, we observed under the same conditions as in Fig. 5 that the gel ruptured instead of buckling (Supplementary Figure 19, Supplementary Movie 10). In these cases, the gel periphery became much denser than the bulk and was apparently much stiffer than in the buckling cases. The exact conditions responsible for this behavior remain to be explored.

How does buckling depend on the system’s mechanical parameters? To answer this question, we estimated these parameters and expressed the buckling wavelength in terms of these. We can estimate the effective drained gel elastic modulus $\kappa = \frac{{4\mu }}{3} + K$ from the relaxation time *τ* by $\kappa \sim \gamma r_{{\mathrm{max}}}^2/\tau$, where *r*_max_ is the radius at *t*_max_ (Supplementary Notes). Using the above expression for *γ* in terms of the solvent viscosity *η* and the pore sizes *ξ*_||_ and *ξ*_⊥_ at *t*_max_, we obtain

3 $$\kappa \sim \eta r_{{\mathrm{max}}}^2/\xi _\parallel \xi _ \bot \tau \sim \eta Rr_{{\mathrm{max}}}h/b\xi _0^2\tau$$

where *R* and *ξ*_0_ are the initial radius and pore size, respectively, and *b* is the final gel thickness. The active stress can be estimated from force balance at steady state, where $Q\zeta {\mathrm{\Delta }}\mu = \kappa \tilde \varepsilon$, where $\tilde \varepsilon$ is the lateral strain at the end of the planar contraction phase relative to the extension of the sheet at *t* = *t*_max_ (Methods). We considered the deforma*t*ion after *t*_max_, because for that phase the exponential decay strongly suggests that *κ* is constant, which is implicit in the last scaling relation. We can now estimate the wavelength *λ*_c_ at buckling onset. For a spatially homogenous activity, the contracting gel is analogous to a system under a compressional external force (Supplementary Notes). For a given external force there exists a minimal extension for which an elastic sheet would buckle (Supplementary Notes). This length was taken as an estimate for the buckling wavelength *λ*_c_. In this way, we get *σ*^act^ = *Qζ*Δ*μ* ~ *κ*(2*πb*/*λ*_c_*)*^2^. With the above expression for the active stress and the effective elastic modulus, we finally obtain

4 $$\lambda _{\mathrm{c}}\sim 2\pi b/\sqrt {\tilde \varepsilon }.$$

**Fig. 6:**
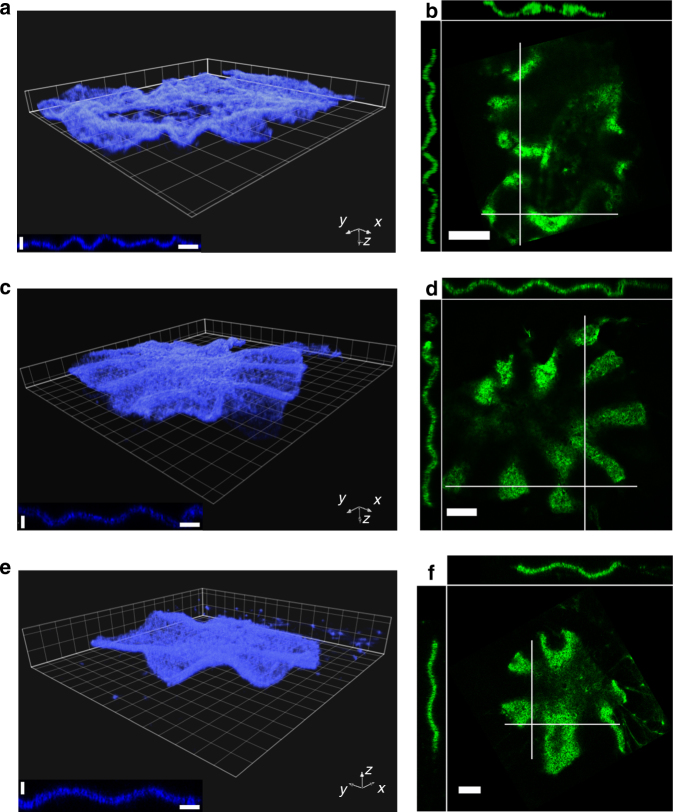
Buckled gels of different thicknesses. **a** Three-dimensional reconstruction of a gel with final sheet thickness *b* = 17 ± 2.5 μm (mean ± SD) from laser scanning confocal fluorescence micrographs. Errors for *b* indicate standard deviation of experimental values of the final gel thickness measured at *N*_thicknesses_ = 93 different places across the gel surface (Methods). **b** Laser scanning confocal fluorescence micrographs of the actin gel in **a** in the *xy* plane (top view), the *xz* plane (top side view), and *yz* plane (left side view). Side views (*xz* and *yz* planes) are measured along the white lines. **c**, **d** as in **a**, **b** but for a gel with *b* = 27 ± 3 μm (mean ± SD, *N*_thicknesses_ = 32). **e**, **f** as in **a**, **b**, but for a gel with *b* = 41 ± 2.5 μm (mean ± SD, *N*_thicknesses_ = 34). Chamber heights *h*: 73 μm (**a**, **b**), 100 μm (**c**, **d**), and 149 μm (**e**, **f**). Initial gel radius *R* and drop volume: 3.4 mm and 2.6 μL (**a**, **b**), 3.25 mm and 3.3 μL (**c**, **d**), and 3.73 mm and 6.5 μL (**e**, **f**). Scale bars: 100 μm (horizontal) and 60 μm (vertical) (insets of **a**, **c**, **e**) and 200 μm (**b**, **d**, **f**). Grid mesh size is 100 μm (**a**, **c**, **e**)

**Fig. 7:**
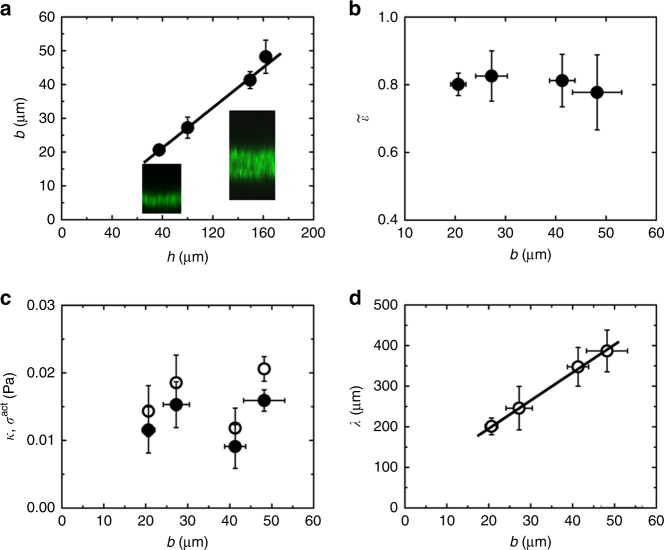
Effect of final sheet thickness *b* on the gel mechanical properties and critical buckling wavelength. **a** Final gel thickness *b* vs. chamber height *h*. **b** Final lateral strain $\tilde \varepsilon$ relative to the extension of the sheet at *t* = *t*_max_ as a function of *b*. **c** Gel effective elastic modulus *κ* (open circles) and active stress *σ*^act^ at the end of contraction (black dots) as a function of *b*. **d** Critical buckling wavelength *λ*_c_ estimated from scaling ($\lambda _{\mathrm{c}} = \frac{{2\pi }}{{\sqrt {\tilde \varepsilon } }}b$, black line) and the experimental buckling wavelength *λ* (open circles) as a function of *b*. Error bars for *b* and *λ* indicate standard deviations for *N*_gels_ gels with *N*_gels_ = 4 for *h* = 77 μm, *N*_gels_ = 3 for *h* = 100 μm, *N*_gels_ = 3 for *h* = 150 μm, and *N*_gels_ = 3 for *h* = 160 μm (Methods). The initial gel radius *R* and drop volume are: 3.4 mm and 2.8 μL for *h* = 77 μm, 3.7 mm and 4.3 μL for *h* = 100 μm, 3.3 mm and 5.3 μL for *h* = 150 μm, and 3.1 mm and 5.0 μL for *h* = 160 μm. Error bars for *κ*, $\tilde \varepsilon$, and *σ*^act^ indicate standard deviations for *N*_gels_ gels with *N*_gels_ = 3 for *h* = 77 μm, *N*_gels_ = 3 for *h* = 100 μm, *N*_gels_ = 4 for *h* = 150 μm, and *N*_gels_ = 3 for *h* = 160 μm (Methods). The initial gel radius *R* and drop volume are: 1.2 mm and 0.37 μL for *h* = 77 μm, 1.3 mm and 0.53 μL for *h* = 100 μm, 1.3 mm and 0.79 μL for *h* = 150 μm, and 1.5 mm and 1.1 μL for *h* = 160 μm. For the straight line in **d**, we took $\tilde \varepsilon = 0.8 \pm 0.02$ (mean ± SD), which was obtained by averaging the strain measured for all gels in **b** (*N*_gels_ = 13), such that *λ*_c_ = (7.0 ± 0.1)*b*

After having established a relation between the buckling wavelength and the final lateral strain, we checked its validity. We started by determining the final thicknesses of gels assembled in chambers of different heights and imaged with confocal microscopy (Fig. 6). Qualitatively, we observed an increase of the final sheet thickness and of the buckling wavelength with the chamber height *h* (Fig. 6). For each chamber height, we investigated 3–4 gels, and found a linear dependence of the final gel thickness *b* on the chamber height *h* (Fig. 7a). In a range of chamber heights between 80 to 160 μm, *b* ≈ *h*/3. As a function of the final sheet thickness *b*, the final strain $\tilde \varepsilon$ varied by less than 10% (Fig. 7b). Similarly, the active stress and the effective elastic modulus *κ* did not depend significantly on the gel thickness (Fig. 7c) showing that in the range of system heights used these are intrinsic material properties that do not depend on the system geometry. Explicitly, we found *κ* ≅ 0.02 Pa. In contrast, an elastic modulus of 10 kPa has been measured for cells^40^. The difference between this value and the one found for our gel is due to the much larger distance between crosslinks in our system (50 μm at *t*_max_) compared to 200 nm in cells^41^. The ratio between the cellular elastic modulus and that of our gel is given by the volume ratio $\xi _{{\mathrm{gel}}}^3/\xi _{{\mathrm{cell}}}^3$, where *ξ*_cell_ and *ξ*_gel_ are the distance between crosslinks in cells and gels, respectively^31^. This explains the factor of 10^6^ between the two moduli. Finally, comparison of the experimental values of the buckling wavelength at steady state *λ* with the values obtained from the scaling relation (Eq. 4) is remarkably good (Fig. 7d). Since $\tilde \varepsilon$ is essentially constant, the wavelength is proportional to the final gel thickness. The value of the strain averaged over all heights, $\tilde \varepsilon = 0.8\, \pm \,0.02$ (mean ± SD, *N*_gels_ = 13), yields $2\pi /\sqrt {\tilde \varepsilon } = 7.0 \pm 0.1$, which agrees very well with the measurements (Fig. 7d). Beyond a gel thickness of 80 μm we did not observe buckling of the gel edges.

As we have shown, initially homogenous elastic sheets contracting under the influence of molecular motors are well-controlled systems to study mechanically induced spontaneous shape transitions in active matter. We found that the network behaves as a poroelastic material where a flow of fluid is generated during contraction. The buckling instability resulted from system self-organization and relied on spontaneous formation of density gradients driven by active contractility.

The detailed conditions for observing buckling still need to be explored. In particular it remains to be seen if the fluid flow is essential for generating strong enough lateral gradients that lead to buckling. Moreover, further experiments are necessary to explore the range of thicknesses for which buckling occurs and how it depends on motor and passive cross-linker concentrations and the lateral extension of the gel.

With respect to applications, it will be interesting to combine our system with recently introduced methods to spatially and temporally structure the activation of myosin motors^8,9^. In this way, it should be possible to generate active origami, where the system contracts into complex, pre-designed three-dimensional structures. By tuning the thickness of the assembly chambers, one can generate growing and actively contracting sheets that can mimic the growth and folding of epithelia. In particular, we anticipate that our experimental system can be used to study the similarity and differences between systems that fold because of differential growth and those that spontaneously fold due to contraction in the absence of growth.

## Methods

### Protein purification

G-actin was purified from rabbit skeletal muscle acetone powder by gel filtration (HiPrep^TM^ 26/60 Sephacryl^TM^ S-300HR, GE Healthcare), stored on ice, and used within 2 weeks. Actin was labeled on Cys374 with Alexa-Fluor 488. Fascin was produced as a GST-fusion protein. Purification of myosin II skeletal muscle was done according to standard protocols^42^. Myosin II was labeled with Alexa-Fluor 568 at pairs of engineered cysteine residues (see details in ref. ^5^).

### Experimental procedure

The motility medium contains 10 mM HEPES pH = 7.0, 1 mM MgCl_2_, 25 mM KCl, an ATP regenerating system (0.5 mg mL^−1^ creatine kinase and 5 mM creatine phosphate), 200 μM EGTA, an anti-bleaching solution (0.1 mg mL^−1^ glucose oxidase, 0.018 mg mL^−1^ catalase, and 5 mg mL^−1^ glucose), 5 μM G-actin, 280 nM fascin, and 16.7 nM of myosin II. The activity of labeled and unlabeled myosin II motors is similar, and they were used at various ratios of 0–100% (labeled/unlabeled).

Network assembly**—**first, myosin II aggregates were prepared by bringing the stock motor solution (at 0.5 M KCl) to the final KCl concentration used in the experiment. Actomyosin network formation is initiated by transferring the preformed motor aggregates into the motility medium (see above). Depending on the experiment, we placed 0.3–7 μL of that solution between a glass slide and a glass coverslip (the exact drop volumes are given for each experiment in the corresponding figure captions). We varied the drop volume to generate circular gels of different diameters. The gel initial diameter was determined by the chamber height *h* and drop volume. To prevent protein adsorption, the glass coverslip and slide were cleaned using piranha solution and coated with an inert polymer (PEG-mal *M*_w_ = 5000 g mol^−1^ (Nanocs)). The effect of viscosity on contraction dynamics was determined by adding glycerol to the motility medium. Specifically we varied the percentage of glycerol from 0 to 34 to obtain an increase in solvent viscosity from *η*_w_ to 2.76*η*_w_ where *η*_w_ is the viscosity of water at 25 °C^43^.

### Cell chamber preparation

Chambers were prepared using a glass slide and a glass coverslip or two glass coverslips. The chambers were sealed and delineated by Teflon, Parafilm, or tape spacers located between a glass slide and a coverslip. The choice of a given type of spacer sets the cell height *h* to a given range. We used various drop volumes to generate actin sheets of different lateral extensions. In our experiments, the chamber height *h* varied between 70 and 250 μm, and the drop radius ranged between 0.12 and 0.5 cm.

### Microscopy techniques

Samples were imaged within 1–2 min after mixing, with an Olympus IX-71 or Leica DMI3000 inverted microscopes. The samples were excited at 488 and 561 nm and the images were recorded simultaneously in two channels using a Dual view Simultaneous Imaging System (Photometrics) with an Andor DV887 EM-CCD camera. Movies overlaying both channels’ acquisitions were created using the Metamorph software (Molecular devices). The 3D structure of the sheet at steady state was determined by laser scanning confocal microscopy. Confocal micrographs of the gel at steady state were collected using Leica SP5 laser scanning confocal system on a DMI6000 microscope and analyzed with the LAS X software (Leica Microsystems). To follow the dynamics of contraction in 3D, we used spinning disk confocal microscopy. We used an Olympus IX-81 inverted microscope, supplemented with an Andor XD system with Andor DU-897 camera and acquisition with Andor IQ software. Analysis of the gel contraction and solvent flow dynamics was performed using Matlab (MathWorks) and Metamorph.

### Edge velocity of contractile actomyosin sheets

The analysis of the lateral contraction velocity (i.e., edge velocity) was performed by measuring the changes in sheet radius (extracted from changes in sheet area), which is possible for gels with a radius of up to 1.5 mm. The sheet radius was determined by first thresholding the fluorescence images. The area of the super-threshold region was then determined. For each velocity plot we extracted the acceleration (*a*), the maximal velocity (*v*_max_), the time at which the maximal velocity was reached (*t*_max_), the lateral decay time *τ*, and final lateral strains *ε* and $\tilde \varepsilon$. Let *R* denote the initial radius of the sheet, *r*_max_ the radius of the sheet at *t* = *t*_max_, and *r*_end_ its radius at the end of the contraction process (*t* = *t*_end_). Then $\varepsilon = \frac{{R - r_{{\mathrm{end}}}}}{R}$ is the lateral strain at the end of the planar contraction phase relative to the initial extension of the sheet at *t* = 0 and $\tilde \varepsilon = \frac{{r_{{\mathrm{max}}} - r_{{\mathrm{end}}}}}{{r_{{\mathrm{max}}}}}$ is the lateral strain at the end of the planar contraction phase relative to the extension of the sheet at *t* = *t*_max_.

The contraction velocity of the gel edge in the *z*-direction was determined by measuring the changes in gel thickness *e* with time. The edge velocity corresponds to changes in the gel half-thickness. The decay time *τ*_z_ was then evaluated by fitting the data with an exponential function.

### Actin and myosin spatial velocity profiles

Particle image velocimetry (PIV) was used to determine the vector velocity fields and velocity profiles of contractile networks. PIV was also used to compare the vector velocity fields of actin (***v***_actin_) and myosin motors (***v***_myosin_). Analysis was performed using Matlab particle image velocimetry (PIV) statistical tool^44^. Velocity vectors calculation was based on a chosen interrogation square window with edge sizes of 40, 32, or 20 pixels with an overlap of 20, 16, or 10 pixels between each window, respectively. Determination of the average particle displacement field was accomplished by computing the spatial auto-correlation of the particle images. The same procedure has been repeated for all images of the contraction movie, providing a description of the network velocity dynamics during contraction.

### Actin velocity-myosin velocity correlation

To obtain the local actin velocity-myosin velocity correlation (Supplementary Figure 3), we considered pairs of velocities of actin and myosin in the gel and determined the angle $\theta$ between them: ${\mathrm{cos\theta }} = \frac{{{\boldsymbol{v}}_{{\mathrm{actin}}} \cdot {\boldsymbol{v}}_{{\mathrm{myosin}}}}}{{\left| {{\boldsymbol{v}}_{{\mathrm{actin}}}} \right|\left| {{\boldsymbol{v}}_{{\mathrm{myosin}}}} \right|}}$, where $\left| {{\boldsymbol{v}}_{{\mathrm{actin}}}} \right|$ and $\left| {{\boldsymbol{v}}_{{\mathrm{myosin}}}} \right|$ are the local actin and myosin speeds (magnitude). The normalized difference between the local actin and myosin velocities was evaluated from: $\frac{{\left| {{\boldsymbol{v}}_{{\mathrm{actin}}} - {\boldsymbol{v}}_{{\mathrm{myosin}}}} \right|}}{{\left| {{\boldsymbol{v}}_{{\mathrm{actin}}}} \right|}}$. This process was performed for all velocity pairs across the gel surface.

### Measuring fluid flow during contraction

The fluid flow was characterized by adding fluorescent beads (200 nm diameter Fluoresbrite YG Microspheres from Polysciences and 2300 nm diameter Nile red beads from Spherotech) to the motility medium. Large beads were used to analyze the fluid flow during the initial and intermediate contraction phases, whereas small beads were used in the final stage of contraction, where the mesh size would have hindered the motion of the large beads. For the experiments, the beads were first incubated with actin monomers and then Bovine Serum Albumin (BSA) to reduce their interaction with the actin network. Particle tracking (Metamorph and Matlab) was used to extract the center-of-mass position of the beads as a function of time.

### Local bead velocity-gel velocity correlation

The bead velocity $${\boldsymbol{v}}_{{\mathrm{bead}}}$$ was calculated by taking the bead’s center-of-mass position at times *t*, $$\left( {x\left( t \right),y\left( t \right)} \right)_{{\mathrm{bead}}}$$ and $$t + {\mathrm{\Delta }}t$$ $$\left( {x\left( {t + {\mathrm{\Delta }}t} \right),y\left( {t + {\mathrm{\Delta }}t} \right)} \right)_{{\mathrm{bead}}}$$, and dividing by $${\mathrm{\Delta }}t$$, that is $${\boldsymbol{v}}_{{\mathrm{bead}}} = \frac{{{\mathrm{\Delta }}{\boldsymbol{r}}_{{\mathrm{bead}}}}}{{{\mathrm{\Delta }}t}} = \frac{{\left( {x\left( {t + {\mathrm{\Delta }}t} \right) - x\left( t \right),y\left( {t + {\mathrm{\Delta }}t} \right) - y\left( t \right)} \right)_{{\mathrm{bead}}}}}{{{\mathrm{\Delta }}t}}$$. To determine the local gel velocity $${\boldsymbol{v}}_{{\mathrm{gel}}}$$, we used defects in the gel or points of filament crossings as fiducial markers. The gel velocity was determined by taking the position at times *t*, $$\left( {x\left( t \right),y\left( t \right)} \right)_{{\mathrm{gel}}}$$, and $$t + {\mathrm{\Delta }}t$$ $$\left( {x\left( {t + {\mathrm{\Delta }}t} \right),y\left( {t + {\mathrm{\Delta }}t} \right)} \right)_{{\mathrm{gel}}}$$, and dividing by $${\mathrm{\Delta }}t$$. Thus, $${\boldsymbol{v}}_{{\mathrm{gel}}} = \frac{{{\mathrm{\Delta }}{\boldsymbol{r}}_{{\mathrm{gel}}}}}{{{\mathrm{\Delta }}t}} = $$$$\frac{{\left( {x\left( {t + {\mathrm{\Delta }}t} \right) - x\left( t \right),y\left( {t + {\mathrm{\Delta }}t} \right) - y\left( t \right)} \right)_{{\mathrm{gel}}}}}{{{\mathrm{\Delta }}t}}$$. To obtain the local bead velocity-gel velocity correlation (Fig. 4c), we considered pairs of velocities of a bead and a trackable nearby point in the gel, e.g., a branching point or a particularly bright spot, and determined the angle $$\theta $$ between them: $${\mathrm{cos}}\theta = \frac{{{\boldsymbol{v}}_{{\mathrm{bead}}} \cdot {\boldsymbol{v}}_{{\mathrm{gel}}}}}{{\left| {{\boldsymbol{v}}_{{\mathrm{bead}}}} \right|\left| {{\boldsymbol{v}}_{{\mathrm{gel}}}} \right|}}$$, where $$\left| {{\boldsymbol{v}}_{{\mathrm{bead}}}} \right| = \frac{{\sqrt {\left( {\left( {x\left( {t + {\mathrm{\Delta }}t} \right) - x\left( t \right)} \right)^2 + \left( {y\left( {t + {\mathrm{\Delta }}t} \right) - y\left( t \right)} \right)} \right)_{{\mathrm{bead}}}^2} }}{{{\mathrm{\Delta }}t}}$$ and $$\left| {{\boldsymbol{v}}_{{\mathrm{gel}}}} \right| = \frac{{\sqrt {\left( {x\left( {t + {\mathrm{\Delta }}t} \right) - x\left( t \right)} \right)^2 + \left( {y\left( {t + {\mathrm{\Delta }}t} \right) - y\left( t \right)} \right)_{{\mathrm{gel}}}^2} }}{{{\mathrm{\Delta }}t}}$$.

### Bead radial velocity *v*_r_

The radial velocity of the beads, *v*_r_ (Fig. 3) was measured by projecting the bead velocity $${\boldsymbol{v}}_{{\mathrm{bead}}}$$ onto the direction, i.e., the unity vector $$\frac{{{\mathrm{\Delta }}{\boldsymbol{r}}_0}}{{\left| {{\mathrm{\Delta }}{\boldsymbol{r}}_0} \right|}}$$, connecting the gel center (*x*_0_, *y*_0_) and the bead center-of-mass position at time *t*. Thus, the radial velocity $$v_{\mathrm{r}} = {\boldsymbol{v}}_{{\mathrm{bead}}} \cdot \frac{{{\mathrm{\Delta }}{\boldsymbol{r}}_0}}{{\left| {{\mathrm{\Delta }}{\boldsymbol{r}}_0} \right|}} = \frac{{\left( {x\left( {t + {\mathrm{\Delta }}t} \right) - x\left( t \right),y\left( {t + {\mathrm{\Delta }}t} \right) - y\left( t \right)} \right)_{{\mathrm{bead}}}}}{{{\mathrm{\Delta }}t}} \cdot $$$$\frac{{\left( {x\left( t \right)_{{\mathrm{bead}}} - x_0,y\left( t \right)_{{\mathrm{bead}}} - y_0} \right)}}{{\left| {{\mathrm{\Delta }}{\boldsymbol{r}}_0} \right|}}$$, where $$\left| {{\mathrm{\Delta }}{\boldsymbol{r}}_0} \right| = \sqrt {\left( {x\left( t \right)_{{\mathrm{bead}}} - x_0} \right)^2 + \left( {y\left( t \right)_{{\mathrm{bead}}} - y_0} \right)^2} $$.

### Density propagation in a contracting gel

Density propagation was determined by following in time the position corresponding to a given value of the gel density as it propagates from the gel boundary inwards. Specifically, we recorded the density profile along a line that went through the center of the gel. For all the recorded time points, we then followed the position of a specific density value along this line. In Fig. 4g, we present the distance *d* traveled in the reference frame of the laboratory as a function of time.

### Pair correlation function *G*(*r*)

The pair correlation function (or radial distribution function) allows one to quantify the structural order in materials and is commonly used to distinguish between gases, liquids, and solids^45^. Specifically, *G*(*r*) provides information about the probability of finding a particle at a radial distance *r* from another particle located at another position, denoted as the origin for convenience. Here, we use it to determine the length scale over which the system can be considered homogenous. For a two-dimensional system the pair correlation function is: $G \left( r \right) = \frac{{\langle{n\left( r \right)}\rangle}}{{2\pi r{\mathrm{\Delta }}r}}\frac{1}{\rho }$, where 〈*n*(*r*)〉 is the mean number of particles at a radial distance *r* from a reference particle located at the origin. *G*(*r*) is normalized by dividing it by the area of a shell with radius *r* and thickness Δ*r*, 2*πr*Δ*r*. Finally, *ρ* is the mean surface density of particles. It serves as a normalization factor such that *G*(*r*) = 1 for a homogenous system. For a system with local order, *G*(*r*) tends to unity above a critical length. We determined the radial density correlation functions for actin and myosin as follows: we first suppressed the background fluorescence by determining those 5% of all pixels with the lowest intensity and setting their intensity to zero. Then for each pixel within the central third of the image and with a non-zero intensity, which we call the reference pixels, we record the number of pixels with a non-zero intensity for a given distance *r*. By averaging with respect to reference pixels, we obtain 〈*n*(*r*)〉. We divide this value by 2*πr*Δ*r*, where we have used Δ*r* *=* 1.6 μm to optimize the statistics. Finally, we divide by the area of all non-zero pixels relative to the area of the full image to get the value of *G*(*r*). In an infinite system this would imply *G(r)* → 1 for *r* → ∞.

### Buckling wavelength *λ*, gel thickness *b*, chamber height *h*

Laser scanning confocal microscopy was used to visualize the 3D structure of the gel sheet at steady state and determine the colocalization of actin and myosin motors within the gel. From the micrographs, we extracted parameters value including: the chamber height *h*, the final sheet thickness *b*, the buckling wavelength *λ* at steady state (as a measure of *λ*_c_), and final vertical strain *ε*_z_. The thickness *b* was evaluated from three confocal cross-sections in the *xz* direction—one passing through the gel center, the others at the two gel boundaries—and three similar cross-sections in the *yz* direction. For each cross section, we measured the gel thickness at equidistant positions along the gel. Typically, 30–90 values were thus extracted for each gel. The error bars for *b* indicate standard deviation of these values. The mean final thickness was then used to calculate the final vertical strain *ε*_z_. Let *h* denote the initial thickness of the sheet and *b* the final thickness of the gel sheet. Then $\varepsilon _{\mathrm{z}} = \frac{{h - b}}{h}$ is the vertical strain at the end of the vertical contraction phase relative to the initial thickness of the sheet. The buckling wavelength *λ* was estimated from the fluorescence intensity along a line tracing the gel periphery in *xy* confocal cross-sections, where we measured the distance between adjacent intensity maxima and between adjacent intensity minima (see Supplementary Figure 8 for example). The measurements of the buckling wavelength were done on gels with an initial radius of at least 3 mm to have several maxima in steady state; typically, 6–12 maxima and 6–12 minima, depending on the gel size and thickness. The positions of the maxima have been obtained by visual inspection. In addition, we looked at cuts through the gel profile in the *xz* and the *yz* directions. We determined the position of the maxima of the profile by visual inspection. Both methods yielded very similar distributions of the buckling wavelength.

### Estimating the gel mesh size

The initial mesh size *ξ*_0_ was estimated directly from the actin images at contraction onset. In a first step, we identified the pores in the network by visual inspection. This was possible, because they were delimited by actin bundles, which were clearly detectable in the fluorescence micrographs. Then, we determined the distances between two pairs of opposing bundles of a mesh and obtained the corresponding mesh size from their geometric mean. After contraction was initiated, the mesh size *ξ* was always determined at the center of the sheet. For each gel, we have measured the size of 55–90 pores.

### Actin bundles contour length *l*_cont_, end-to-end distance *l*_end-to-end_

We considered actin bundles were limited by branching or by crosslinking points (see Supplementary Figure 4). To determine the contour length of a bundle (*l*_cont_), we traced the latter manually and used the ‘Region Measurements’ function of Metamorph. To determine the end-to-end distance (*l*_end-to-end_), we measured the distance between the two end points of a bundle.

### Estimating the values of *κ*, *σ*^act^, and *λ*_c_

The values of the gel effective elastic modulus are $\kappa = \frac{{\eta Rr_{{\mathrm{max}}}h}}{{b\xi _0^2}\tau}$ (Eq. 3), active stress $\sigma ^{{\mathrm{act}}} = \kappa \tilde \varepsilon$, and wavelength at buckling onset $\lambda _{\mathrm{c}} = \frac{{2\pi }}{{\sqrt {\tilde \varepsilon } }}b$ (Eq. 4) (see main text). For a single gel, the estimation of $\kappa$ required the values of *b*, $\xi _0$, $R$, $r_{{\mathrm{max}}}$, $h$, and *τ*. The value of *b* used in the expression for $\kappa$ was the average of the 30–90 values taken at different positions across the gel surface, see above in Buckling wavelength *λ*, gel thickness *b*, chamber height *h*’. Also, we used the average values of $\xi _0$ for each gel, see above. Finally, the values of *R*, $r_{{\mathrm{max}}}$, and *τ* were evaluated as explained above in ‘Edge velocity of contractile actomyosin sheets’. The value of the initial sheet thickness *h* was extracted from laser confocal micrographs. For a single gel, the value of the active stress $\sigma ^{{\mathrm{act}}}$ was calculated from $\kappa$ and $\tilde \varepsilon$, where $\tilde \varepsilon$ is the lateral strain at the end of the planar contraction phase relative to the extension of the sheet at $t = t_{{\mathrm{max}}}$ (see definition in ‘Edge velocity of contractile actomyosin sheets’). In Fig. 7, the values of $b$, $\lambda$, $\kappa$, $\tilde \varepsilon$, and $\sigma ^{{\mathrm{act}}}$ were averaged over 3-4 gels that were analyzed for each chamber height *h*; error bars indicate the standard deviations for these 3–4 gels. Note that for the calculation of $\kappa$, $\tilde \varepsilon$, and $\sigma ^{{\mathrm{act}}}$, we used small gels (*R* < 1.5 mm), whereas *b* and *λ* were measured on large gels (*R* > 3 mm). The coefficient linking $\lambda _{\mathrm{c}}$ and *b* (Fig. 7d) was obtained from $\lambda _{\mathrm{c}} = \frac{{2\pi }}{{\sqrt {\tilde \varepsilon } }}b$, where we took the value of $\tilde \varepsilon$ averaged over the gels for all initial heights *h* ($N_{{\mathrm{gels}}} = 13$ gels).

### Data availability

The data that support the findings of this study are available from the corresponding author upon reasonable request.

## Electronic supplementary material

Supplementary Information

Description of Additional Supplementary Information

Supplementary Movie 1

Supplementary Movie 2

Supplementary Movie 3

Supplementary Movie 4

Supplementary Movie 5

Supplementary Movie 6

Supplementary Movie 7

Supplementary Movie 8

Supplementary Movie 9

Supplementary Movie 10

Supplementary Movie 11

Supplementary Movie 12

Supplementary Movie 13
