# Supplementary material for: Spontaneous buckling of contractile poroelastic actomyosin sheets
Source: Nat Commun. 2018 Jun 25;9:2461. doi: 10.1038/s41467-018-04829-x (PMC6018151; doi:10.1038/s41467-018-04829-x)
Supplement: Supplementary file 1 — Supplementary Information [file 41467_2018_4829_MOESM1_ESM.pdf]

Spontaneous buckling of poroelastic actomyosin sheets

Ideses et al

Supplementary Notes - Theoretical analysis

Supplementary Table 1

Supplementary Figures 1-19

Supplementary References

Supplementary Notes - Theoretical analysis

Dynamic equations for actively contracting elastic gels

In this section, we present the dynamic equations for an active elastic gel that incorporates the catch bond character of molecular motors, where we will employ the approach developed in Ref. (*1*) for active gels. It provides a phenomenological account of the gel dynamics on large length and time scales and is based on conservation laws and symmetries.

We consider an (isotropic) elastic gel that is permeated by a solvent. We assume that there are no external forces acting on the system and that the elastic properties of the gel in absence of active processes are captured by linear elasticity theory with (constant) bulk and shear moduli κ and μ. In the gel, processes driven by the hydrolysis of Adenosine-Triphosphate generate mechanical stresses. Gradients in these stresses can lead to deformations of the gel that are described by the field $\mathbf{u}$, which gives the displacement of a volume element initially placed at position $\mathbf{r}$. Deformations of the gel will then generate a flow in the permeating solvent, which is described by the solvent velocity field $\mathbf{v}$. The respective densities ρ_{gel} and ρ_{sol} of the gel and the solvent evolve according to the continuity equations

$$\partial_t \rho_{\mathrm{gel}} + \partial_\alpha \rho_{\mathrm{gel}} \dot{u}_\alpha = 0 \tag{1}$$

$$\partial_t \rho_{\mathrm{sol}} + \partial_\alpha \rho_{\mathrm{sol}} v_\alpha = 0. \tag{2}$$

Here, $\dot{\mathbf{u}} \equiv \partial_t \mathbf{u}$ is the gel deformation velocity, $\alpha = x$, y, and z, and we have used the Einstein convention, which implies summation over identical indices.

To first order in the displacement field, the density ρ_{gel} can be expressed in terms of the initial gel density ρ_0 at $t = 0$ and the displacement field $\mathbf{u}$ through

$$\rho_{\mathrm{gel}} = \rho_0 \left(\frac{1}{1 + \partial_\alpha u_\alpha} + \mathcal{O}(u^2) \right), \tag{3}$$

which solves Eq. (1). Furthermore, incompressibility of the solvent yields $\partial_\alpha v_\alpha = 0$.

The displacement and velocity fields are determined by momentum conservation. Since the dynamics is overdamped, inertial terms can be neglected. We consider force balance separately for the gel and the solvent and then use constitutive equations for the partial stresses. The same dynamic equations also follow from considering the multicomponent system close to thermodynamic equilibrium (*2, 3*). We write

$$-\partial_\beta \sigma^{\mathrm{sol}}_{\alpha\beta} = \gamma(\rho_{\mathrm{gel}})\left(\dot{u}_\alpha - v_\alpha\right) \tag{4}$$

$$-\partial_\beta \sigma^{\mathrm{gel}}_{\alpha\beta} = -\gamma(\rho_{\mathrm{gel}})\left(\dot{u}_\alpha - v_\alpha\right). \tag{5}$$

Here, γ describes friction between the gel and the solvent and has units of viscosity divided by a length squared. In general, it depends on the gel density ρ_{gel}. The constitutive equations for the respective stresses are given by linear stress-strain rate and stress-strain relations:

$$\sigma^{\mathrm{sol}}_{\alpha\beta} = 2\eta v_{\alpha\beta} + p\delta_{\alpha\beta} \tag{6}$$

$$\sigma^{\mathrm{gel}}_{\alpha\beta} = \sigma^{\mathrm{el}}_{\alpha\beta} + \sigma^{\mathrm{act}}_{\alpha\beta}. \tag{7}$$

In Equation (6), $v_{\alpha\beta} = \left(\partial_\alpha v_\beta + \partial_\beta v_\alpha\right)/2$ is the symmetric part of the strain-rate tensor, η the solvent's (shear) viscosity, and p its hydrostatic pressure. The pressure is determined by the incompressibility condition. The gel's stress consists of an elastic part and an "active" part generated by the molecular motors that are driven by the hydrolysis of ATP. We write

$$\sigma^{\mathrm{el}}_{\alpha\beta} = \kappa u_{\gamma\gamma}\delta_{\alpha\beta} + 2\mu\left(u_{\alpha\beta} - \frac{1}{d}\delta_{\alpha\beta}u_{\gamma\gamma}\right) \tag{8}$$

$$\sigma^{\mathrm{act}}_{\alpha\beta} = -Q\zeta\Delta\mu\,\delta_{\alpha\beta}. \tag{9}$$

The expression for σ^{el} is the conventional linear relation between the stress and the strain $u_{\alpha\beta} = \left(\partial_\alpha u_\beta + \partial_\beta u_\alpha\right)/2$, where κ denotes the bulk and μ the shear modulus. We take the active stress σ^{act} to be linear in the difference $\Delta\mu$ of the chemical potentials of ATP, Adenosine-Diphosphate (ADP), and inorganic phosphate $\mathrm{P_i}$, which drives the motor activity, and d is the

spatial dimension. On a molecular level, the active stress is mostly a consequence of the action of molecular motors that convert the chemical energy released during ATP hydrolysis into mechanical work. To a good approximation the active stress is thus given by the density of force dipoles generated by the molecular motors (*4*). Here, we take a phenomenological approach and expand the active stress up to linear order in $\Delta\mu$, where ζ is a phenomenological constant.

To account for the catch bond character of the molecular motors, we have introduced *ad hoc* in Eq. (9) the fraction Q of crosslinking motor complexes. It is a dynamic quantity and evolves according to

$$\partial_t Q = k_{\text{on}} \left(1 - Q\right) - k_{\text{off}} \left(\sigma^{\text{el}}_{\alpha\alpha}\right) Q \tag{10}$$

The effective rates k_{on} and $k_{\text{off}} \left(\sigma^{\text{el}}_{\alpha\alpha}\right)$ describe changes in crosslinking through motor-binding and -unbinding, respectively. The catch bond property leads to a reduction of motor unbinding with increasing stress. We write

$$k_{\text{off}} = k^0_{\text{off}} \exp\left\{- \left|\sigma^{\text{el}}_{\alpha\alpha}\right| / \sigma_0 \right\}, \tag{11}$$

where σ_0 is a characteristic stress of the system. Note, that we neglect any contribution of shear stress to the catch bond behavior and consider the effects on motor contractility to be isotropic. – This completes the dynamic equations for an actively contracting elastic gel permeated by a viscous solvent.

Constant active stress

We will now consider the contraction dynamics for a slab of an acto-myosin gel for a constant and homogenous active stress. In that case, the effect of the active stress is equivalent to the application of forces to the gel surfaces. Intuitively, one can understand this by noting that the myosin motors generate force dipoles and that these dipoles cancel in a bulk macroscopic

average. Only at the surfaces this cancellation does not occur and the gel is consequently contracted by a constant surface stress. We also neglect the dependence of the friction coefficient γ on the gel density ρ_{gel}. In this situation, the dynamic equations are linear and can be solved analytically.

Let the gel have extensions $2L_x \times 2L_y \times 2h$ with $L_x, L_y \gg h$ and let it be oriented with its long axes along the x- and y-directions such that the origin coincides with the gel's center of mass. We first write the dynamic equations for the solvent permeating the gel. From Equations (4) and (6) we get (5)

$$\eta \partial_\beta^2 v_\alpha + \partial_\alpha p + \gamma \left(\dot{u}_\alpha - v_\alpha \right) = 0. \tag{12}$$

These equations can be solved for the solvent velocities as functions of the gel displacements. The gel is much thinner than its extension in the lateral dimensions. We therefore expect that the velocity and displacement fields vary only slightly as a function of z. To lowest order, we can obtain a consistent solution by neglecting the dependence of v_x, v_y, u_x, u_y, and p on z, and by assuming $u_z, v_z \propto z$.

We rewrite the dynamic equations by performing a Fourier transform in the lateral direc-

tions. The Fourier components $V_\alpha(\mathbf{q},t)$, $U_\alpha(\mathbf{q},t)$, and $P(\mathbf{q},t)$ are defined through

$$v_x = \sum_{\mathbf{q}} V_x \sin q_x x \cos q_y y$$
$$v_y = \sum_{\mathbf{q}} V_y \cos q_x x \sin q_y y$$
$$v_z = z \sum_{\mathbf{q}} V_z \cos q_x x \cos q_y y$$
$$u_x = u_x^s + \sum_{\mathbf{q}} U_x \sin q_x x \cos q_y y$$
$$u_y = u_y^s + \sum_{\mathbf{q}} U_y \cos q_x x \sin q_y y$$
$$u_z = u_z^s + z \sum_{\mathbf{q}} U_z \cos q_x x \cos q_y y$$
$$p = \sum_{\mathbf{q}} P \cos q_x x \cos q_y y$$

with the wave vector $\mathbf{q} = (q_x, q_y)$ and where u_α^s denote the steady state solutions to the dynamic equations. The solution to Eq. (12) is then

$$V_x = \frac{q_y^2 \dot{U}_x - q_x q_y \dot{U}_y - q_x \dot{U}_z}{q^2 \left(1 + q^2\eta/\gamma\right)}$$
$$V_y = \frac{q_x^2 \dot{U}_y - q_y q_x \dot{U}_x - q_y \dot{U}_z}{q^2 \left(1 + q^2\eta/\gamma\right)}$$
$$V_z = \frac{\dot{U}_z}{1 + q^2\eta/\gamma}$$

and

$$P = \gamma \frac{q_x \dot{U}_x + q_y \dot{U}_y + \dot{U}_z}{q^2}, \tag{13}$$

where we have also used the incompressibility condition $\partial_\alpha v_\alpha = 0$.

From Equations (5) and (7)-(9) with $d = 3$ we find

$$\mu \partial_\beta^2 u_\alpha + \left(\kappa + \frac{\mu}{3}\right) \partial_\alpha \partial_\beta u_\beta = \gamma(\dot{u}_\alpha - v_\alpha)$$

with boundary conditions

$$\sigma_{\alpha\beta}^{\rm tot} = 0$$

at $x = \pm L_x$, $y = \pm L_y$, and $z = \pm h$ since there is no external force applied to the gel. The steady state solution is

$$u_\alpha^s = \frac{1}{3\kappa} Q\zeta\Delta\mu x_\alpha,$$

which can be expressed in terms of the Young's modulus E and the Poisson ratio ν as

$$u_\alpha^s = (1-2\nu)\frac{Q\zeta\Delta\mu}{E} x_\alpha,$$

because $\kappa = E/\left(3(1-2\nu)\right)$. For $Q\zeta\Delta\mu < 0$ it describes a contracted state.

The deviations from the steady state as determined by the Fourier components U_α evolve according to

$$\gamma\left(1-\frac{\gamma q_y^2}{(\gamma+\eta q^2)\,q^2}\right)\dot{U}_x + \frac{\gamma^2 q_x q_y}{(\gamma+\eta q^2)\,q^2}\dot{U}_y + \frac{\gamma^2 q_x}{(\gamma+\eta q^2)\,q^2}\dot{U}_z = - \left(\mu q^2 + \left(\kappa+\frac{\mu}{3}\right) q_x^2\right) U_x - \left(\kappa+\frac{\mu}{3}\right) q_x q_y U_y - \left(\kappa+\frac{\mu}{3}\right) q_x U_z \qquad (14)$$

$$\frac{\gamma^2 q_y q_x}{(\gamma+\eta q^2)\,q^2}\dot{U}_x + \gamma\left(1-\frac{\gamma q_x^2}{(\gamma+\eta q^2)\,q^2}\right)\dot{U}_y + \frac{\gamma^2 q_y}{(\gamma+\eta q^2)\,q^2}\dot{U}_z = - \left(\kappa+\frac{\mu}{3}\right) q_y q_x U_x - \left(\mu q^2 + \left(\kappa+\frac{\mu}{3}\right) q_y^2\right) U_y - \left(\kappa+\frac{\mu}{3}\right) q_y U_z \qquad (15)$$

$$\gamma\left(1-\frac{\gamma}{\gamma+\eta q^2}\right)\dot{U}_z = -\mu q^2 U_z. \qquad (16)$$

The boundary conditions impose $q_x = (2n+1)\pi/(2L_x)$ and $q_y = (2m+1)\pi/(2L_y)$ with integers n and m.

The solution is of the form $U_\alpha = \sum_i U_{\alpha,i}\exp(-\omega_i t)$, where, in lowest order in q^2, two rates ω_i are, respectively, $\omega_f = \mu/\eta$ and $\omega_s = (4\mu/3+\kappa)q^2/\gamma$. The mode associated with ω_f relaxes fast and governs relaxation in z-direction. The other mode is diffusive in nature as it decays as q^2 for $q \to 0$ and thus relaxes slowly in the (x, y)-direction. The constants $U_{\alpha,i}$ characterize the

various modes up to a scaling factor that is determined from the initial conditions. We choose $u_\alpha(\mathbf{r}, t = 0) = 0$.

Contraction in presence of a dynamic active stress for a circular symmetric sheet

In the main text, we analyse the situation, where the active stress can change with time and space and where the friction coefficient depends on the gel density. In this case, an analytical solution is typically not available and we turn to numerical solutions of the dynamic equations. Consider a disk-shaped network. This situation corresponds to times, when contraction in z-direction is completed. In this case, we do not need to account for derivatives of the displacement field or of the solvent velocity in the z-direction. Assuming invariance of the disk with respect to rotations around the disk center, we solve the dynamic equations in cylindrical coordinates. The relevant components of the stress in the gel are given by

$$\sigma_{rr}^{\text{gel}} = \kappa\left(\partial_r u_r + \frac{u_r}{r}\right) + 2\mu\partial_r u_r - Q\zeta\Delta\mu \tag{17}$$

$$\sigma_{\theta\theta}^{\text{gel}} = \kappa\left(\partial_r u_r + \frac{u_r}{r}\right) + 2\mu\frac{u_r}{r} - Q\zeta\Delta\mu. \tag{18}$$

The dynamics of the fraction of bound motors Q is again given by Eqs. (10) and (11). As we will see below, we do not need the expressions for the stress in the solvent, because v_r can be eliminated from the dynamic equations with the help of the continuity equation. The dynamic equations read

$$-\gamma\left(\dot{u}_r - v_r\right) = -\left(\nabla\cdot\sigma^{\text{gel}}\right)_r \tag{19}$$

$$= -\left[\partial_r\sigma_{rr}^{\text{gel}} + \frac{1}{r}\left(\sigma_{rr}^{\text{gel}} - \sigma_{\theta\theta}^{\text{gel}}\right)\right] \tag{20}$$

To determine the radial component of the solvent velocity v_r, we use the continuity equation for the total mass density $\rho_{\text{tot}} = \rho_{\text{gel}} + \rho_{\text{sol}}$:

$$\frac{1}{r}\partial_r\left(r\phi\dot{u}_r + r\left(1-\phi\right)v_r\right) = 0, \tag{21}$$

where $\phi = \rho_{\text{gel}}/\rho_{\text{tot}}$ is the gel volume fraction. In terms of the initial gel volume fraction ϕ_0, it is given by

$$\phi = \frac{\phi_0}{(1 + \partial_r u_r)\,(1 + u_r/r)}. \tag{22}$$

Since the total material flux at the boundary of the system equals zero, the continuity equation yields

$$v_r = -\frac{\phi}{1-\phi}\dot{u}_r. \tag{23}$$

With this result, we obtain one partial differential equation for the radial component of the displacement vector field u_r:

$$\dot{u}_r = \frac{1-\phi}{\gamma}\left[\partial_r \sigma_{rr}^{\text{gel}} + \frac{1}{r}\left(\sigma_{rr}^{\text{gel}} - \sigma_{\theta\theta}^{\text{gel}}\right)\right] \tag{24}$$

$$= \frac{1-\phi}{\gamma}\partial_r\left[(\kappa + 2\mu)\left\{\partial_r u_r + \frac{u_r}{r}\right\} - Q\zeta\Delta\mu\right]. \tag{25}$$

This equation is complemented by the boundary conditions. At the center, we have $u_r\,(r=0) = 0$. We consider the situation, where there are no forces applied to the border of the gel. Since the border moves in time the corresponding boundary conditions read $\sigma_{rr}^{\text{gel}}\,(r = R + u_r(R)) = 0$ and $\sigma_{\theta\theta}^{\text{gel}}\,(r = R + u_r(R)) = 0$. Here, R is the initial and $R + u_r(R)$ the current radius of the gel.

Dimensionless form of the dynamic equations and parameter values. For the numeric solution of the partial differential equation (25) we scale all lengths with a tenth of the initial radius $R/10$, time with $\gamma R^2/(100\kappa)$, and stresses with κ. The dynamic equation for u_r then has two dimensionless parameters, namely μ/κ and $\zeta\Delta\mu/\kappa$. In addition, the solution depends on the initial volume fraction ϕ of the gel, the rates of motor attachment and detachment k_{on} and k_{off}^0, respectively, and the characteristic stress σ_0, Eq. (11).

For the calculations presented in the main text, the parameter values with dimensions are given in Supplementary Table 1. We chose $\kappa = \mu$, because the Poisson ratio $\nu \approx 0$, see Figure 2b, which implies $2\mu \approx 3\kappa$. For these values, the length scale is $150\,\mu$m and the time scale is 18.75 s.

Initial condition and dynamics of Q in the numerical solutions. We start the numerical solution in the fully relaxed state, that is $u_r(r) = 0$ for all $0 \leq r \leq R$ and in absence of any motor activity, that is $Q(r) = 0$ for all $0 \leq r \leq R$. To mimic the reorganization phase, we keep Q spatially homogenous and increase its value linearly with time to the equilibrium value given by the attachment-detachment dynamics, Eq. (10), in absence of elastic stresses, that is

$$Q(r,t) = \frac{k_{\text{on}}}{k_{\text{on}} + k_{\text{off}}^0} \frac{1}{T_{\text{reorg}}} t \tag{26}$$

for $0 \leq t \leq T_{\text{reorg}}$, where T_{reorg} is the duration of the reorganization phase. For larger times, Q follows the dynamics of Eq. (10).

Numerical solution of the dynamic equations. Using dimensionless variables and parameters as introduced above, the dynamic equations read

$$\dot{u}_r = (1-\phi)\,\partial_r \left[(1+2\mu)\left\{\partial_r u_r + \frac{u_r}{r}\right\} - Q\zeta\Delta\mu\right] \tag{27}$$

$$\partial_t Q = k_{\text{on}}\,(1-Q) - k_{\text{off}}^0 \exp\left\{-\left|\sigma_{\alpha\alpha}^{\text{el}}\right|/\sigma_0\right\} Q \tag{28}$$

For the numerical solution of the dynamic equations we use an explicit Euler forward scheme. To discretize the spatial derivatives on the right hand side, we introduce two different lattices. One lattice is fixed with sites at $i\Delta r$, $i = 0, \ldots, N$ with $N = R/\Delta r$. The discretized velocity for the solvent v_i gives the solvent velocity at $i\Delta r$. The second lattice carries the gel and is dynamic. Its sites are given by $i\Delta r + u_i$, $i = 0, \ldots, N$, where u_i is the radial displacement vector at $i\Delta r$. At $t = 0$ both lattices coincide. The gel volume fraction and the stresses are

associated with the lattice bonds between the lattice sites, and will be, respectively, denoted by $\phi_{i-\frac{1}{2}}$, $\sigma_{rr,i-\frac{1}{2}}$, and $\sigma_{\theta\theta,i-\frac{1}{2}}$ for the bond between sites i and $i-1$. From Equation (22) we have for $i = 2, \ldots, N$

$$\phi_{i-\frac{1}{2}} = \phi_0 \frac{(2i-1)\,\Delta r^2}{[(2i-1)\,\Delta r + u_i + u_{i-1}]\,[\Delta r + u_i - u_{i-1}]} \tag{29}$$

and

$$\phi_{\frac{1}{2}} = \phi_0 \frac{\Delta r^2}{(\Delta r + u_1)^2}. \tag{30}$$

For the stresses we find follwoing Eqs. (17) and (18):

$$\sigma_{rr,i-\frac{1}{2}} = (1+2\mu)\left(\frac{u_i - u_{i-1}}{\Delta r}\right) + \frac{u_i + u_{i-1}}{(2i-1)\,\Delta r} - Q_{i-\frac{1}{2}}\zeta\Delta\mu \tag{31}$$

$$\sigma_{\theta\theta,i-\frac{1}{2}} = \left(\frac{u_i - u_{i-1}}{\Delta r}\right) + (1+2\mu)\,\frac{u_i + u_{i-1}}{(2i-1)\,\Delta r} - Q_{i-\frac{1}{2}}\zeta\Delta\mu \tag{32}$$

for $i = 2, \ldots, N$ and

$$\sigma_{rr,\frac{1}{2}} = (1+2\mu)\,\frac{u_1}{\Delta r} + \frac{u_1}{\Delta r} - Q_{\frac{1}{2}}\zeta\Delta\mu \tag{33}$$

$$\sigma_{\theta\theta,\frac{1}{2}} = \frac{u_1}{\Delta r} + (1+2\mu)\,\frac{u_1}{\Delta r} - Q_{\frac{1}{2}}\zeta\Delta\mu \tag{34}$$

$$\sigma_{rr,N+\frac{1}{2}} = \sigma_{\theta\theta,N+\frac{1}{2}} = 0. \tag{35}$$

Then, the time evolution is given by

$$\dot{u}_i = \left(1 - \frac{\phi_{i-\frac{1}{2}} + \phi_{i+\frac{1}{2}}}{2}\right)\left\{\frac{\sigma_{rr,i+\frac{1}{2}} - \sigma_{rr,i-\frac{1}{2}}}{\Delta r + \frac{u_{i+1}+u_{i-1}}{2}} + \frac{1}{2}\frac{\sigma_{rr,i+\frac{1}{2}} + \sigma_{rr,i-\frac{1}{2}} - \sigma_{\theta\theta,i+\frac{1}{2}} - \sigma_{\theta\theta,i-\frac{1}{2}}}{i\Delta r + u_i}\right\} \tag{36}$$

or $i = 1, \ldots, N$ if we set $u_0 = u_{N+1} = 0$.

Time evolution. In Supplementary Figures 15-18 we present the dynamics of various quantities. During the contraction process, the gel volume fraction ϕ is clearly maximal at the gel

boundary. This illustrates that contraction starts from the boundary. Contraction proceeds down to 10% of the original radius such that the final, homogenous gel density is $\phi_\infty = 100\phi_0$, where ϕ_0 is the initial gel volume fraction.

Similarly to the gel volume fraction, the elastic stress first increases at the gel boundary and then propagates towards the center of the circular gel sheet, Supplementary Figure 16. In the steady state, both, the radial elastic stress $\sigma_{rr}^{\mathrm{el}}$ and the tangential elastic stress $\sigma_{\theta\theta}^{\mathrm{el}}$ are homogenous.

The profile of the fraction of bound motors Q is essentially homogenous throughout the contraction process, Supplementary Figure 17. It increases with time and eventually reaches 1. For the chosen parameters, even a comparatively small elastic stress will essentially suppress all motor unbinding.

Finally, the velocity profiles for the gel contraction velocity as well as for the fluid velocity resemble nicely the experimental data, Supplementary Figure 18. Notably, towards the end of the contraction process, the solvent flow speed exceeds the gel's contraction speed as found experimentally.

The wavelength at the onset of buckling

For a thin elastic sheet of thickness b compressed from the side, the relation between the critical buckling stress σ_c and the buckling wavelength λ_c is given by $\sigma_c = \kappa(2\pi b)^2/\lambda_c^2$, which follows from equating the compression and bending energy terms (*6*). For the critical buckling stress, we take the estimate of the active stress.

Supplementary Table

parameter	value
κ	0.05 Pa
μ	0.05 Pa
$\zeta\Delta\mu$	-0.18 Pa
η	0.05 Pa s
ξ_{gel}^2	1200 μm^2
$\gamma = \eta\xi^{-2}$	$1.5 \cdot 10^{-4}$ Pa s μm^{-2}
k_{on}	0.27 s^{-1}
k_{off}^0	5.1 s^{-1}
σ_0	10^{-4} Pa
T_{reorg}	23.4 s

Supplementary Table 1: Values of the parameters used in the numerical calculations.

Supplementary Figures

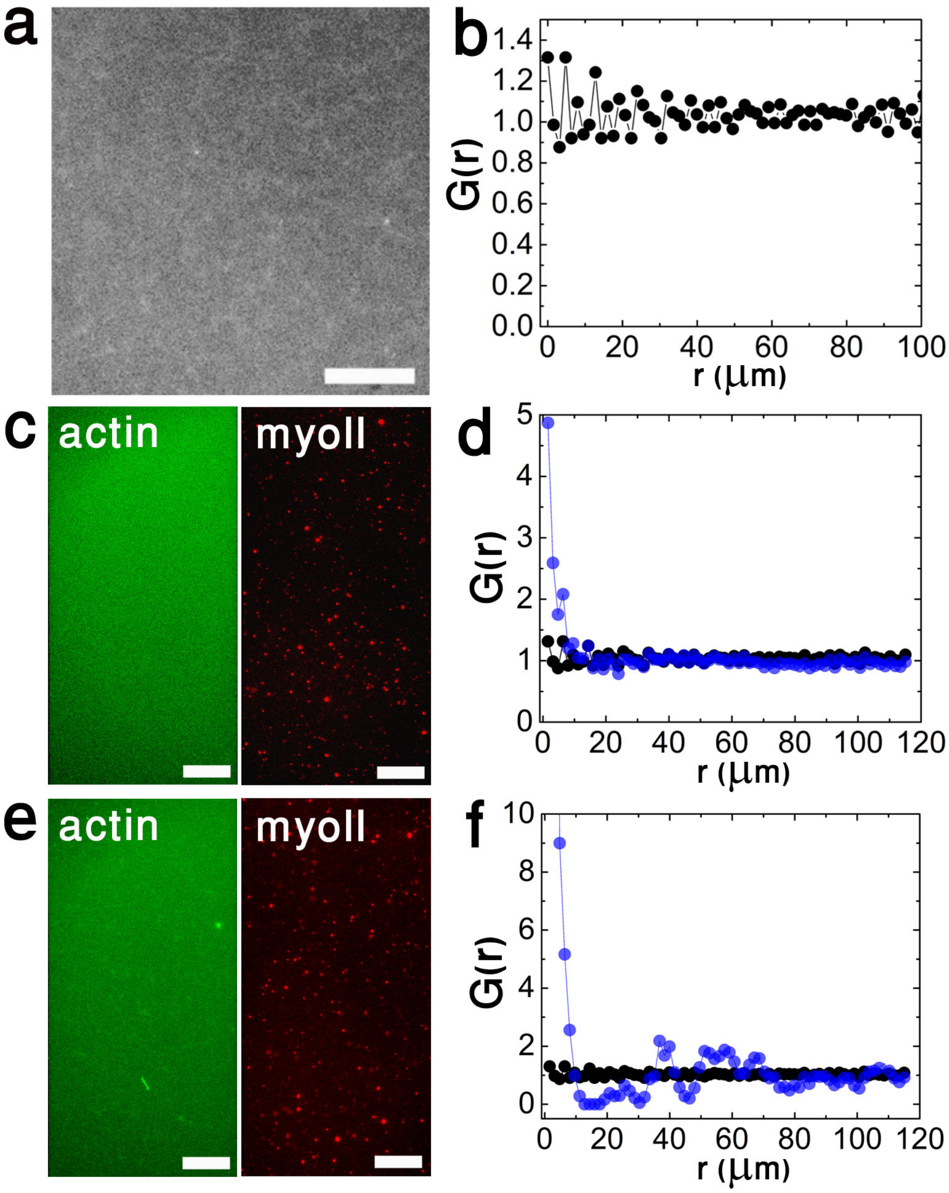

Supplementary Figure 1: The gel is homogenous in phase (i). a) Fluorescence image of actin labeled with Alexa-Fluor 488 and b) corresponding density correlation function $G(r)$ for the gel shown in Figure 1b at $t = 200$ s. c) Fluorescence images of actin labeled with Alexa-Fluor 488 and myosin labeled with Alexa-Fluor 568 at time $t = 151$ s and d) corresponding density correlation functions for actin (black) and myosin (blue) for the gel shown in Figure 1a. e,f) As in (c,d) but for $t = 195$ s. Scale bars: 100 μm.

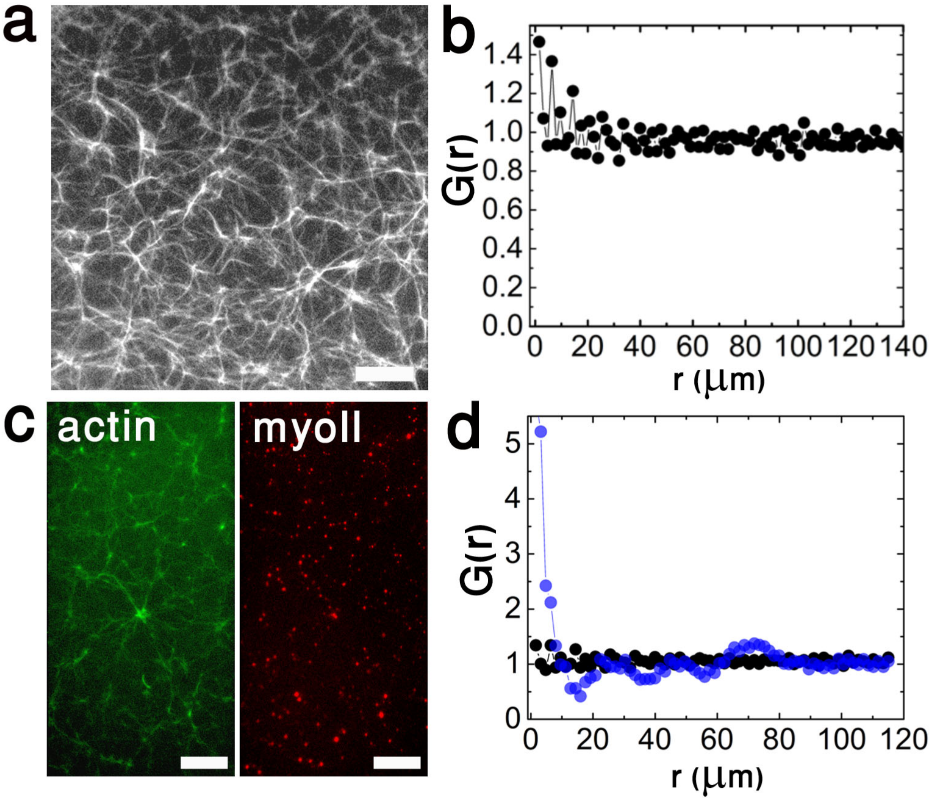

Supplementary Figure 2: The gel is homogenous on scales larger than the mesh size in phase (ii). a) Fluorescence image of actin labeled with Alexa-Fluor 488 and b) corresponding density correlation function $G(r)$ for the gel shown in Figure 1b at $t = 308$ s, which is the time just before macroscopic contraction of the gel was detectable. c) Fluorescence images of actin labeled with Alexa-Fluor 488 and myosin labeled with Alexa-Fluor 568 and d) corresponding density correlation functions for actin (black) and myosin (blue) for the gel shown in Figure 1a at $t = 285$ s, that is, at contraction onset. Scale bars: 100 μm.

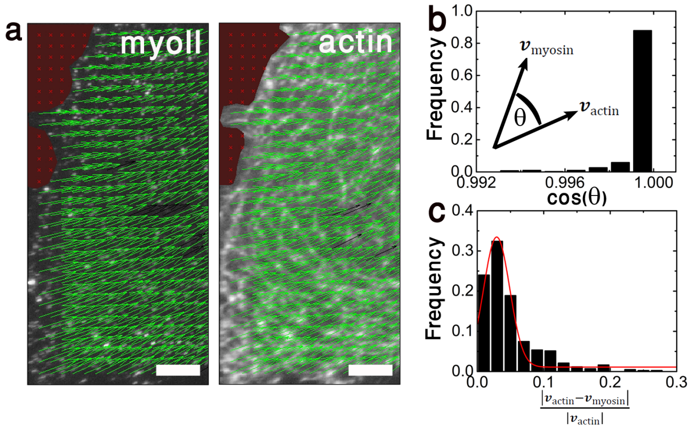

Supplementary Figure 3: Myosin clusters move with the gel during the contraction phase. a) Velocity field (green arrows) of the myosin (left) and the actin (right) obtained from particle image velocimetry. Scale bars: 100 μm. b) Distribution of cosines of the angle between the actin and myosin velocities at the same locations. c) Distribution of the normalized differences between actin and myosin speeds at the same positions, $|\boldsymbol{v}_{\text{actin}} - \boldsymbol{v}_{\text{myosin}}| / |\boldsymbol{v}_{\text{actin}}|$. The gel is the same as in Figure 1a.

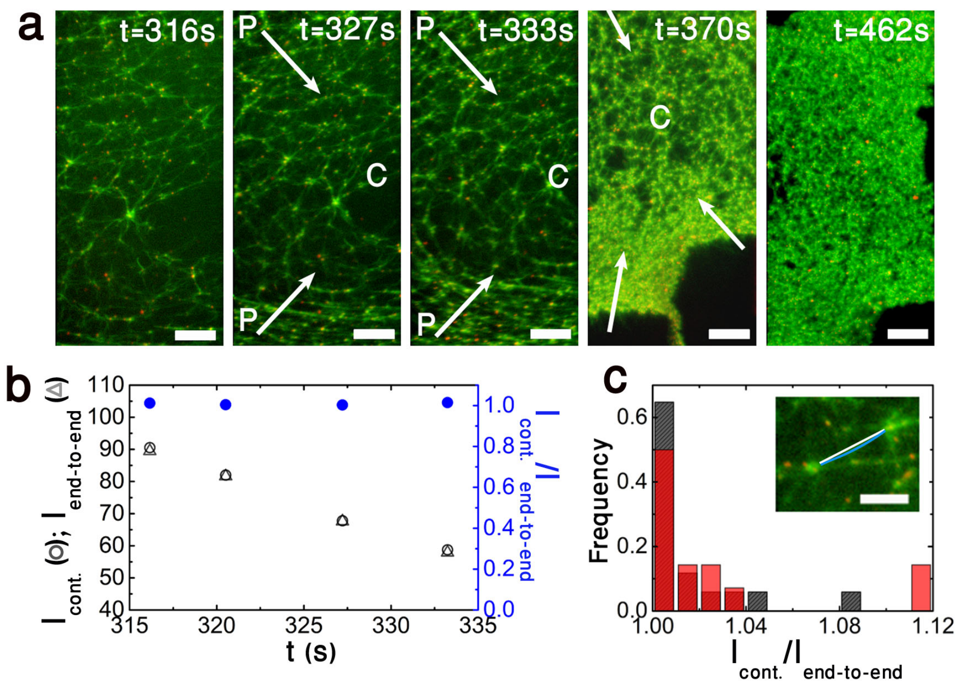

Supplementary Figure 4: Filament bundles remain straight during the contraction process. a) Fluorescent micrographs of the gel in Figure 1a. Actin is labeled with Alexa-Fluor 488 (green) and myosin with Alexa-Fluor 568 (red). Contraction starts from the periphery P towards the center C. Consequently, the gel density, which is inversely proportional to the network mesh size cube, displays a gradient during the contraction process at times $t = 316$ s, 327 s, 333 s, and 370 s. Initially ($t = 285$ s, see Supplementary Figure 2c,d) and in steady state ($t \geq 462$ s), the density is homogenous. Scale bars: 100 μm. b) Contour length l_{cont} (circles) and end-to-end distance $l_{\text{end-to-end}}$ (triangles) of bundles as well as the ratio of the two lengths (blue) of a filament bundle as a function of time. c) Distribution of the ratio between the contour length and end-to-end distance for $N_{\text{bundles}} = 15$ bundles at $t = 316$ s (solid red) and $t = 327$ s (striped gray). Inset: contour (blue) and straight line connecting the ends (white) of a typical bundle ($t = 316$ s). Scale bar: 50 μm.

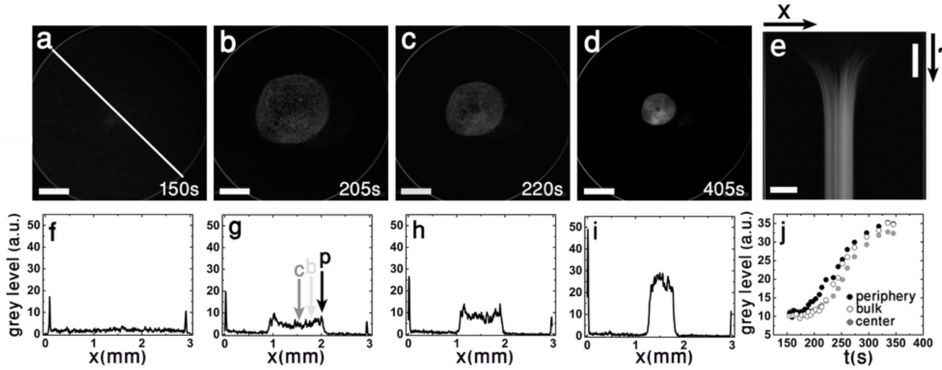

Supplementary Figure 5: Density fields of a contracting sheet that does not buckle. a-d) Subsequent fluorescence micrographs of a contracting circular acto-myosin sheet. Actin was labeled with Alexa-Fluor 488. Scale bar: 500 μm. e) Kymograph of the density along the line indicated in (a). Scale bar: 500 μm and time scale bar: 1 min. f-i) Density profiles along the line indicated in (a) and corresponding to (a-d). j) Density as a function of time for the three points – gel periphery, center, and bulk (at half-length from sheet edge) – indicated in (g). Starting from the same value, the densities at these points differ during the contraction process. Eventually, they saturate at the same value, indicating that the gel has become homogenous again. This is the same gel shown in Figure 1e-h.

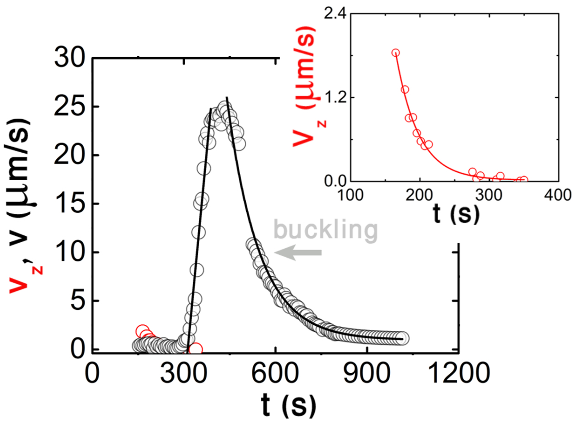

Supplementary Figure 6: Contraction velocity of the gel presented in Figure 2a-c. Black: lateral contraction velocity obtained by tracking a bright fluorescent spot close to the gel boundary, which is close to the velocity at which the sheet radius changes; red: half gel-thickness velocity obtained from confocal fluorescence microscopy. Inset: zoom in for v_z. The initial linear increase in v_z was not accessible with our setup. The lines represent linear and exponential fits to the data with slope $a = 0.16 \pm 0.006$ μm/s^2 and characteristic times $\tau = 105 \pm 4$ s and $\tau_z = 32 \pm 2$ s. Buckling occurs at advanced stages of contraction ($t = 580$ s corresponding to $1.5\ \tau$ after $t_{\rm max}$, where $t_{\rm max}$ is reached at 430 s).

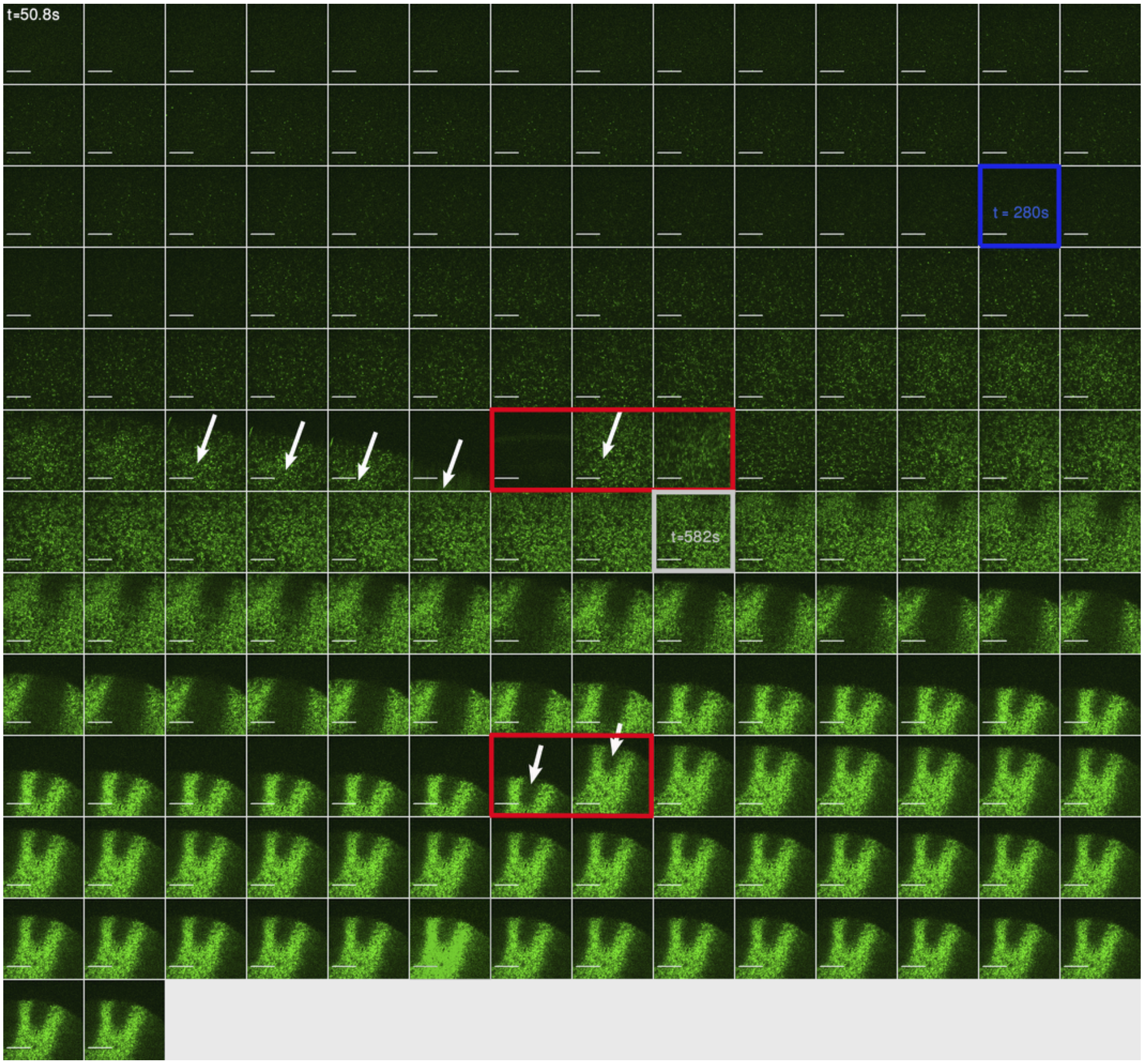

Supplementary Figure 7: Subsequent snapshots of the contracting and buckling gel shown in Figure 2a-c. Confocal micrographs obtained with a spinning disk microscope for a fixed z-position of a region close to the boundary of a contracting sheet with actin labeled by Alexa-Fluor 488. Contraction in the z-direction occurs prior to lateral contraction, which starts at $t = 280$ s (blue frame). At $t = 582$ s the gel starts to buckle at the boundary (grey frame) as is evident from the developing bright and dark stripes that emerge perpendicular to the boundary. White arrows indicate the direction of contraction, red frames indicate time points at which the region was shifted to follow the contracting boundary. Scale bars: 160 μm.

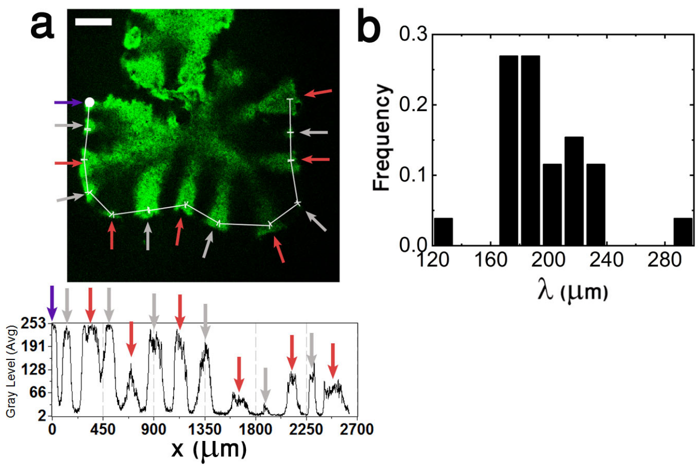

Supplementary Figure 8: Distribution of buckling wavelengths for the gel shown in Figure 2d,e. a) Confocal section obtained with a laser scanning microscope at a specific height z. White lines connect maxima in the fluorescence intensity shown below corresponding to extrema of the buckles that are indicated by arrows. The violet arrow marks the beginning of the line. Scale bar: 200 μm. b) Distribution of the distances between adjacent intensity maxima and between adjacent intensity minima ($N_{\mathrm{wavelengths}} = 24$, $N_{\mathrm{gels}} = 1$) corresponding to the buckling wavelength λ.

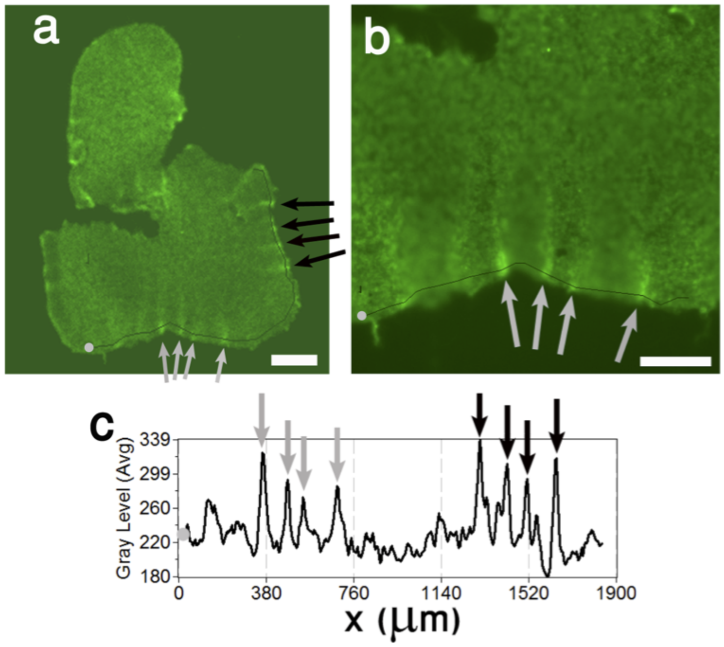

Supplementary Figure 9: Buckles appear as bright stripes at low magnification. a, b) Epifluorescence micrographs of the gel shown in Figure 2d,e. Scale bars: 150 μm (a) and 100 μm (b). c) Fluorescence intensity profile along the lines indicated in (a). The arrows point to intensity maxima that are due to gel buckling.

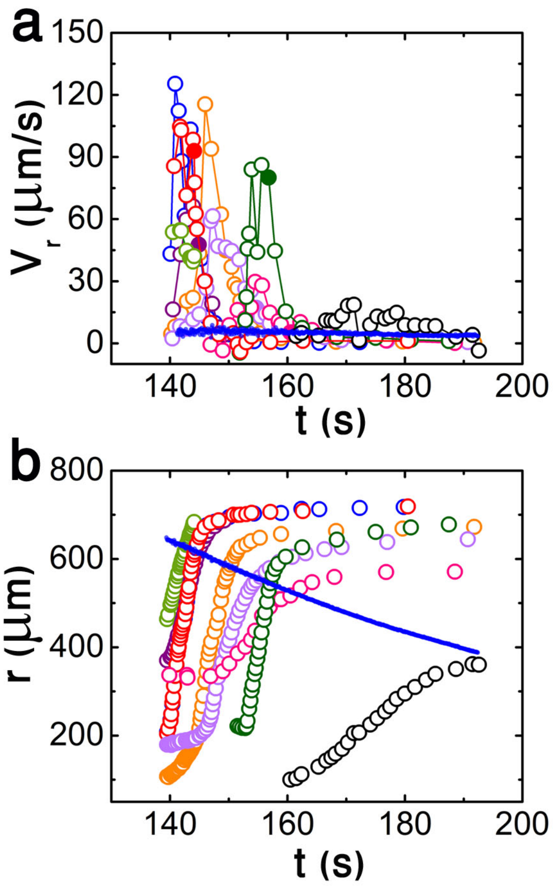

Supplementary Figure 10: Analysis of the trajectories of fluorescent beads, which are immersed in the solvent as the gel contracts, from the gel in Figure 3, see Supplementary Movie 7. Part of the data are presented in Figure 3. a) Radial velocity v_r of 9 beads as a function of time (open circles). The filled circles indicate the time points, when the beads left the gel. The blue dots indicate the radial velocity of the gel boundary. b) Distance from the gel center of the same beads as a function of time (open circles). The blue dots indicate the gel radius. The bead diameter was 2.3 μm.

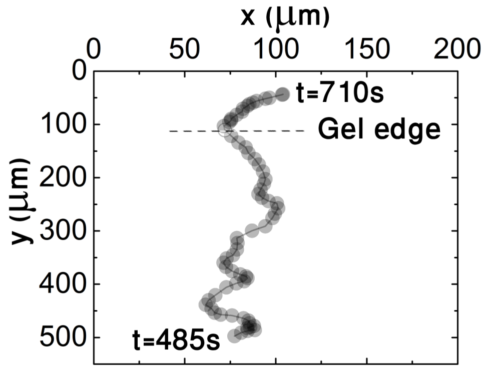

Supplementary Figure 11: Trajectory of the bead shown in Figure 4a, which is immersed in the solvent as the gel contracts. In the field shown, the gel contracts on average towards to bottom. The coordinates are with respect to the origin of the camera. The bead diameter was 200 nm.

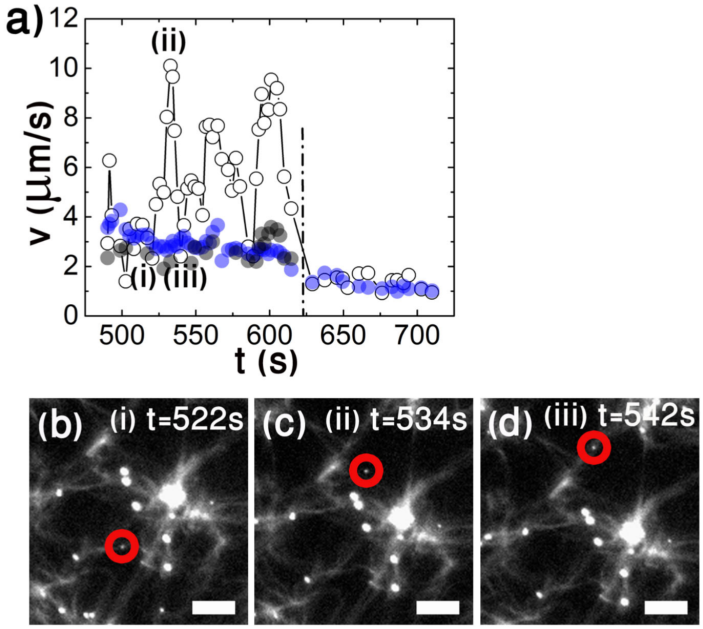

Supplementary Figure 12: Movement of the bead (diameter 200 nm) shown in Figure 4a. a) Local bead speed (open circles), local gel speed (grey circles), and edge speed of the gel (blue circles) as a function of time. b-d) Snapshots indicate the position of the bead with respect to the enveloping pore for selected times. The bead is marked by a red circle. Scale bar: 50 μm. Local bead and gel speeds: same data as in Figure 4b. Speeds were recorded towards the end of the contraction process.

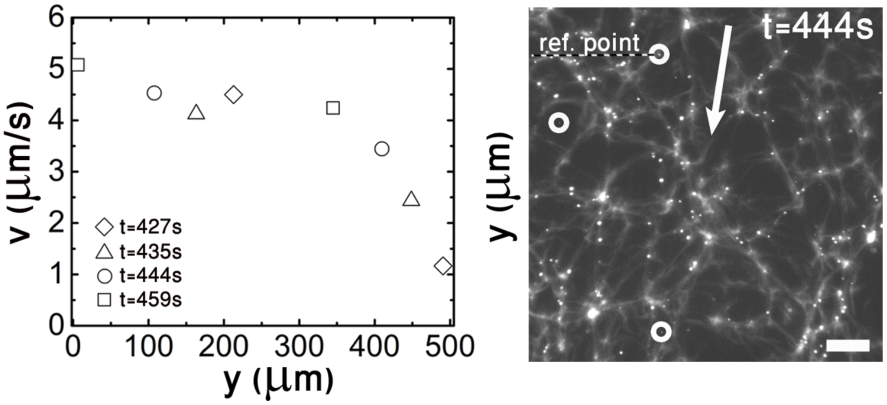

Supplementary Figure 13: Speed of two beads (diameter: 200 nm) immersed in the fluid penetrating the gel shown in Figure 4a obtained by tracking the beads. The bead position is determined as the vertical distance from a reference point that moves with the contracting gel. The bead speeds were determined only at time points when both beads were located well away from actin bundles in the middle of a pore. Data were obtained towards the end of the contraction process. Left: speeds of the two beads as a function of the vertical distance to the reference point for different time points. The speed of the bead closer to the boundary is always higher than that of the other bead. The difference in the speeds decreases with time. Right: snapshot of the beads. Beads fluoresce at the same wavelength of actin labelled by Alexa-Fluor 488. Many beads have accumulated in actin bundles. The circles indicate the tracked beads and the reference point, the arrow points into the direction of gel contraction. Scale bar: 100 μm.

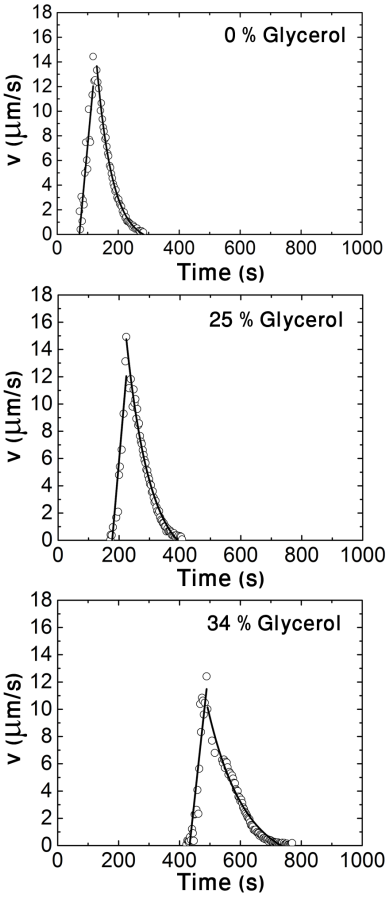

Supplementary Figure 14: Gel contraction velocity as a function of time for different solvent viscosities. The viscosity was changed by adding glycerol to the solvent. The viscosities were $\eta = 2.09\eta_w$ (25% Glycerol) and $2.76\eta_w$ (34%), where η_w is the viscosity of water at 25°C (*7*). The lines indicate linear and exponential fits to the data with slopes a and characteristic times τ of $a = 0.27 \pm 0.03$ μm/s^2 and $\tau = 51.2 \pm 1.5$ s (0% Glycerol), $a = 0.26 \pm 0.03$ μm/s^2 and $\tau = 70.2 \pm 3.5$ s (25% Glycerol), and $a = 0.21 \pm 0.02$ μm/s^2 and $\tau = 120 \pm 7.6$ s (34% Glycerol).

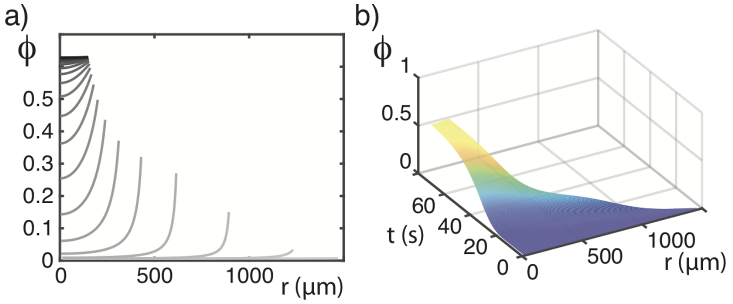

Supplementary Figure 15: a) Gel volume fraction as a function of r for time points $t_i = i * 4.7$ s, with $i = 1, \ldots, 20$. Density increases with increasing time. b) Gel volume fraction as a function of r and t.

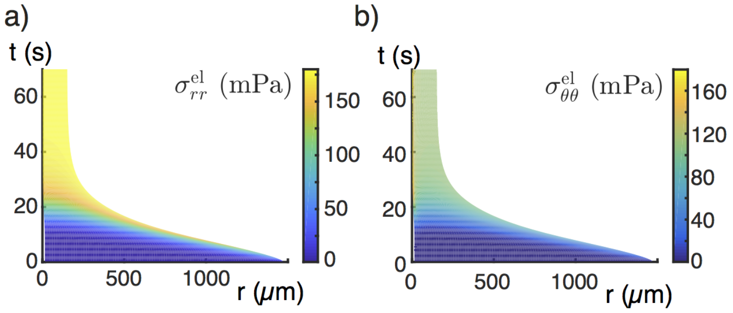

Supplementary Figure 16: Elastic stress in the gel as a function of r and t. a) $\sigma_{rr}^{\mathrm{gel}}$, b) $\sigma_{\theta\theta}^{\mathrm{gel}}$. Although the tangential stress lags somewhat behind the radial stress, they eventually have the same value.

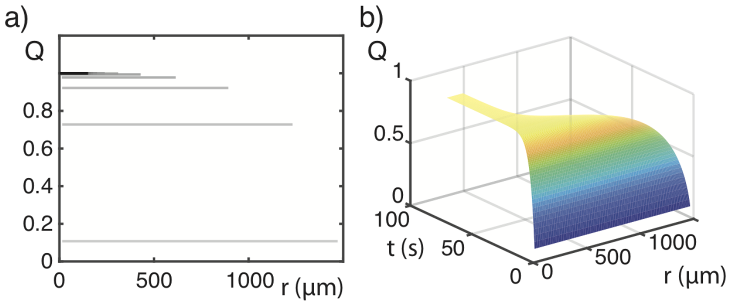

Supplementary Figure 17: a) Fraction of bound motors Q as a function of r for time points $t_i = i * 4.7$ s, with $i = 1, \ldots, 20$. The value of Q increases with increasing time. b) Fraction of bound motors Q as a function of r and t.

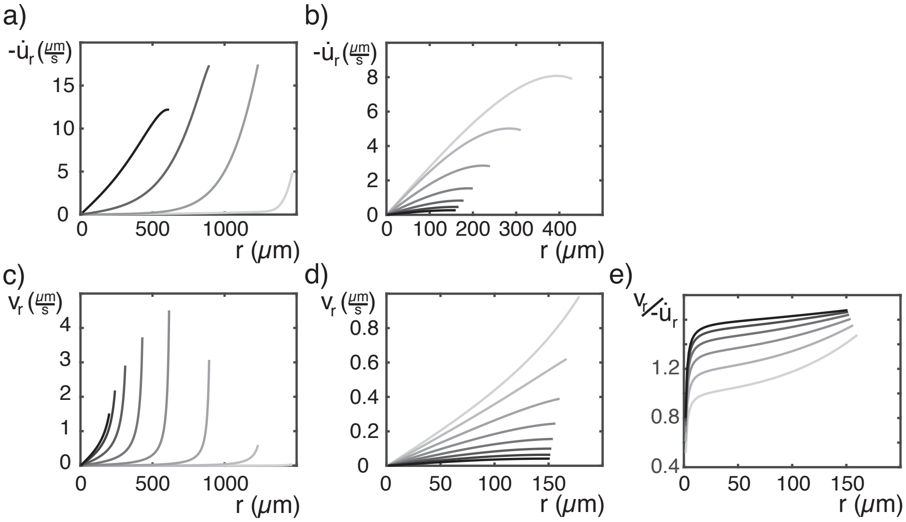

Supplementary Figure 18: Profiles of the gel contraction velocity $\dot{u}_r$ and of the solvent velocity v_r for various time points. a) Gel contraction velocity for times t =0, 4.7, 9.4, 14.1 s. b) Gel contraction velocity for times t =18.8, 23.4, …, 46.8 s. c) Solvent velocity for times t =0, 4.7, …, 32.8 s. d) t =37.5, 42.2, …, 70.3 s. e) Ratio of the solvent velocity and the gel contraction velocity for times t =46.8 s, 51.5 s, …, 70.3 s. In the late stages of contraction, the solvent moves thus faster than the gel. For all panels later times are indicated by darker shades of grey. The time of the maximal velocity is $t_{\max} = 7$ s.

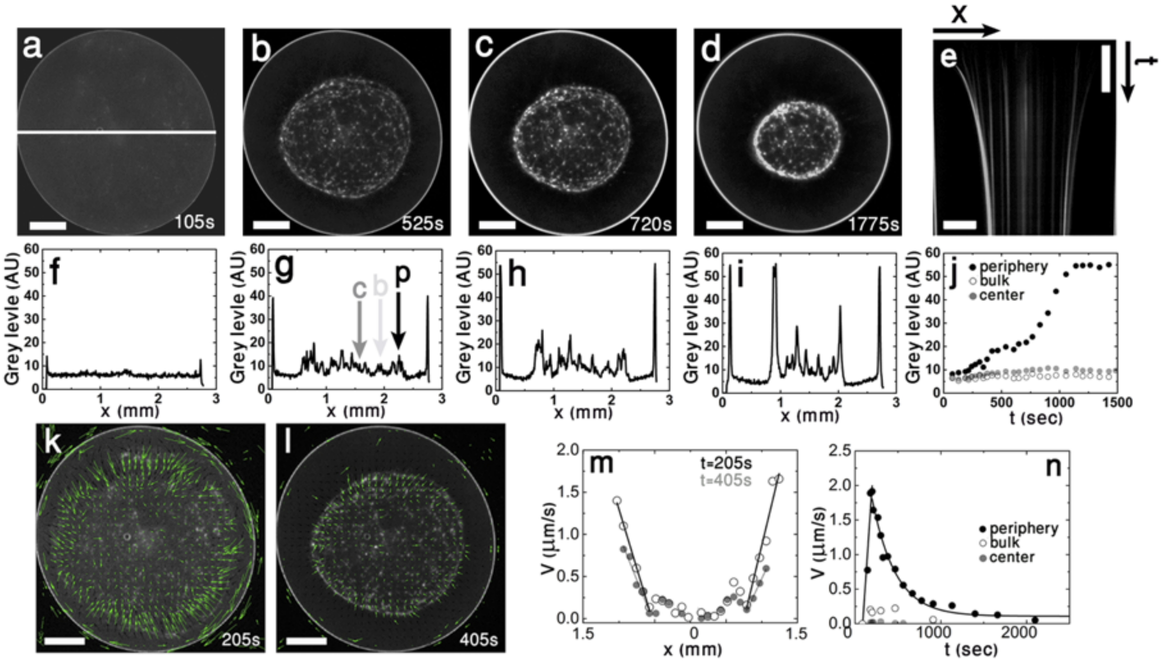

Supplementary Figure 19: Density fields of a contracting sheet that does not buckle. a-d) Subsequent fluorescence micrographs of a contracting circular acto-myosin sheet. Actin was labeled with Alexa-Fluor 488. e) Kymograph of the density along the line indicated in (a). f-i) Density profiles along the line indicated in (a) and corresponding to (a-d). j) Density as a function of time for the three points – gel periphery, center, and bulk (at half-length from sheet edge) – indicated in (g). Starting from the same value, the densities at these points differ during the contraction process and in the steady state. k,l) Snapshots of velocity fields (green arrows) obtained from particle image velocimetry. Contraction clearly starts at the gel boundary. m) Velocity profiles along the line indicated in (a). n) Velocity as a function of time for the three points indicated in (g). Actin is labeled with Alexa-Fluor 488. Scale bars: 500 μm. Scale bar time: 5min (e).

Supplementary References

1. Kruse, K., Joanny, J.-F., Jülicher, F., Prost, J. & Sekimoto, K. Asters, vortices, and rotating spirals in active gels of polar filaments. *Phys. Rev. Lett.* **92**, 078101 (2004).

2. Joanny, J.-F. , Jülicher, F., Kruse, K. & Prost, J. Hydrodynamic theory for multi-component active polar gels. *New J. Phys.* **9**, 422 (2007).

3. Callan-Jones, A. C. & Jülicher, F. Hydrodynamics of active permeating gels. *New J. Phys.* **13**, 093027 (2011).

4. Zemel, A, Rehfeldt, F., Brown, A. E. X., Discher, D. E. & Safran, S. A. Optimal matrix rigidity for stress-fibre polarization in stem cells. *Nat. Phys.* **6**, 468-473 (2010).

5. Yuval, J. & Safran, S. A. Dynamics of elastic interactions in soft and biological matter. *Phys. Rev. E* **87**, 042703 (2013).

6. L. D. Landau, E. M. Lifshitz, *Theory of Elasticity*, vol. 7 of *Course of Theoretical Physics* (Butterworth-Heinemann Ltd, 2004).

7. Cheng, N.-S. Formula for the Viscosity of a Glycerol?Water Mixture. *Ind. Eng. Chem. Res.* **47**, 3285-3288 (2008).
