# Supplementary material for: Spontaneous buckling of contractile poroelastic actomyosin sheets
Source: Nat Commun. 2018 Jun 25;9:2461. doi: 10.1038/s41467-018-04829-x (PMC6018151; doi:10.1038/s41467-018-04829-x)
Supplement: Supplementary file 2 — Description of Additional Supplementary Information [file 41467_2018_4829_MOESM2_ESM.pdf]

Description of Additional Supplementary Files

File Name: Supplementary Movie 1
Description: Fluorescence imaging of an actomyosin network at phases one and two of polymerization and reorganization, respectively, at high magnification (top view). Actin is labeled with Alexa-Fluor 488. This is the same gel as in Figure 1b-d.

File Name: Supplementary Movie 2
Description: Simultaneous fluorescence imaging of the formation and contraction of an actomyosin sheet (top view). Myosin motor clusters labeled with AlexaFluor 568 appear on the left and actin labeled with Alexa-Fluor 488 on the right. This is the same gel as shown in Figure 1a, i and in Supplementary Figures 1-4.

File Name: Supplementary Movie 3
Description: Contraction initiates at the gel boundaries. Particle Image Velocimetry analysis of a contracting circular network at low magnification (top view). Actin is labeled with Alexa-Fluor 488. This is the same gel as in Figure 1e-h.

File Name: Supplementary Movie 4
Description: Spinning disc confocal imaging of an actin sheet that spontaneously buckles. Maximal projection is shown in the xz-plane. Actin is labeled with Alexa-Fluor 488. This is the same gel as in Figure 2a-c.

File Name: Supplementary Movie 5
Description: Three views of the same gel shown in Supplementary Movie 4. Top view is the xy-plane for a specific height, left side view is the yz-plane, and top side view is the xz-plane. The xz- and yz-planes are taken along the lines shown in Figure 2c.

File Name: Supplementary Movie 6
Description: Laser scanning confocal imaging of the 3D gel structure shown in Fig. 2d, e at steady state. Actin is labeled with Alexa-Fluor 488.

File Name: Supplementary Movie 7
Description: The contracting actomyosin gel behaves as a poroelastic material I (low magnification). Beads of diameter 2.3 µm (right) are moving on average in the outward radial direction as the gel (left) contracts towards its center. Actin is labeled with Alexa-Fluor 488. Beads are Nile-red. This is the same gel as in Figure 3 and in Supplementary Figure 10.

File Name: Supplementary Movie 8
Description: The contracting actomyosin gel behaves as a poroelastic material II (high magnification). Beads of diameter 0.2 µm (the circle shows one such bead) move on average into the outward radial direction as the gel contracts towards its center. Actin is labeled with Alexa-Fluor 488. Beads are Fluoresbrite Yellow-Green. Scale bar is 100 µm. This is the same gel as in Figure 4a-c and in Supplementary Figures 11-13.

File Name: Supplementary Movie 9
Description: Fluorescence imaging of a buckling contracting acto-myosin sheet at low magnification (top view). Actin is labeled with Alexa-Fluor 488. This is the same gel as in Figure 5.

File Name: Supplementary Movie 10
Description: Fluorescence imaging of a contracting acto-myosin sheet that undergoes rupture at low magnification (top view). Actin is labeled with Alexa-Fluor 488. This is the same gel as in Supplementary Figure 19.

File Name: Supplementary Movie 11
Description: Laser scanning confocal image of the 3D gel structure shown in Figure 6a, b at steady state. Actin is labeled with Alexa-Fluor 488. Grid mesh size is 100 µm.

File Name: Supplementary Movie 12
Description: Laser scanning confocal image of the 3D gel structure shown in Figure 6c, d at steady state. Actin is labeled with Alexa-Fluor 488. Grid mesh size is 100 µm.

File Name: Supplementary Movie 13
Description: Laser scanning confocal image of the 3D gel structure shown in Fig. 6e, f at steady state. Actin is labeled with Alexa-Fluor 488. Grid mesh size is 100 µm.
